# Cell surface topology creates high Ca^2+^ signalling microdomains

**DOI:** 10.1016/j.ceca.2010.01.005

**Published:** 2010-04

**Authors:** Jens Christian Brasen, Lars Folke Olsen, Maurice B. Hallett

**Affiliations:** a CelCom, Institute of Biochemistry and Molecular Biology, University of Southern Denmark, Denmark; b Neutrophil Signalling Group, School of Medicine, Cardiff University, UK

**Keywords:** Microdomains, Mathematical model, Calcium, Ca^2+^

## Abstract

It has long been speculated that cellular microdomains are important for many cellular processes, especially those involving Ca^2+^ signalling. Measurements of cytosolic Ca^2+^ report maximum concentrations of less than few micromolar, yet several cytosolic enzymes require concentrations of more than 20 μM Ca^2+^ to be activated. In this paper, we have resolved this apparent paradox by showing that the surface topology of cells represents an important and hitherto unrecognized feature for generating microdomains of high Ca^2+^ in cells. We show that whereas the standard modeling assumption of a smooth cell surface predicts only moderate localized effects, the more realistic “wrinkled” surface topology predicts that Ca^2+^ concentrations up to 80 μM can persist within the folds of membranes for significant times. This intra-wrinkle location may account for 5% of the total cell volume. Using different geometries of wrinkles, our simulations show that high Ca^2+^ microdomains will be generated most effectively by long narrow membrane wrinkles of similar dimensions to those found experimentally. This is a new concept which has not previously been considered, but which has ramifications as the intra-wrinkle location is also a strategic location at which Ca^2+^ acts as a regulator of the cortical cytoskeleton and plasma membrane expansion.

## 1 Introduction

The ability to restrict enzyme activation to sub-domains within the cell is crucial for cell behaviour, such as migration, directed pseudopodia formation and cell polarization. Although calpains are known to be important in these activities [1–3] since they are relatively non-specific proteases, their unrestricted activation would wreak havoc within the cell. As these enzymes are only activated by very high Ca^2+^ concentrations, i.e. concentrations that are much higher than reached within the bulk cytosol, the activation signals must be restricted to strategic locations within the cell. While the existence of high Ca^2+^ microdomains within cells has long been discussed [1–3], theoretical considerations have suggested that this level of Ca^2+^ can only exist transiently in limited cytosolic space within 100 nm off the open mouth of Ca^2+^ influx channels [2,3]. However, these models have been based on topological smooth cell surfaces, rather than more realistic micro-topologies which often include irregular surface wrinkles.

The surface of cells is rarely smooth and often appears wrinkled when viewed with sufficient resolution, such as scanning electron microscopy or atomic force microscopy [4,5]. Typical non-tissue cells, such as neutrophils, macrophages, lymphocytes and mast cells [5,6], have multiple cells wrinkles, which when viewed by transmission microscopy appear as microvilli. These wrinkles are permanent or semi-permanent structures which have a specific spectrum of surface molecules, such as integrins and selectins on neutrophil [7] and lymphocytes [8] and are sub-light microscopic, being about 100 nm wide and projecting 800 nm from the cell surface [6]. In macrophages and neutrophils, these wrinkles act as a membrane reservoir for the “expansion” of the cell surface area during phagocytosis [9,10] and spreading [11]. The cytosolic free Ca^2+^ signal which accompanies these events [12,13] permits the unwrinkling of the membrane and involves activation of the Ca^2+^ dependent protease calpain-1 [10,11,13] which probably cleaves proteins such as talin and ezrin [14] that hold the wrinkles in place. As the concentration of Ca^2+^ required for calpain-1 activation is at least 2 orders of magnitude higher than the resting level of cytosolic free Ca^2+^, i.e. 10–50 μM [15–17], this activation signal must clearly be restricted to strategic locations within the cell. Experimentally, transient Ca^2+^ puffs can be observed within the bulk cytosol as Ca^2+^ is released from storage sites within a number of non-excitable cell types [18–20]. However, the cytosolic free Ca^2+^ concentration reached is within the physiological range of 0.1–1 μM similar to that in the bulk cytosol during Ca^2+^ influx. Nevertheless, the existence of high Ca^2+^ microdomains near the plasma membrane has long been suspected [1,2] and apparently physiological secretion of secretory granules seem to require high (50–100 μM) Ca^2+^ concentrations [21], suggesting that high cytosolic free Ca^2+^ is generated physiologically. Recently, it has been shown that TRPM2 channel opening is also activated by high micromolar cytosolic Ca^2+^ concentrations [22,23].

However, theoretical models [1–3] based on smooth spherical (or other shaped) surfaces have suggested that this level of Ca^2+^ can only exist transiently very near the open mouth of Ca^2+^ influx channels (within 100 nm). Therefore only molecules very close to open channels would sense such signals. Since it is speculated that high Ca^2+^ would be strategically important within wrinkles, we therefore sought to construct a model which included a wrinkled cell surface in order to establish whether the wrinkled topology had a significant influence on the near membrane Ca^2+^ concentration during Ca^2+^ influx. We show here that the wrinkled surface of cells provides a mechanism for generating high Ca^2+^ domains where the concentration of Ca^2+^ reaches tens of micromolar while the bulk cytosol remains sub-micromolar and that any cell with a wrinkled surface topology can have high Ca^2+^ microdomains due to this effect. These anatomical structures provide a hitherto unrecognised mechanism for restricting the activation of Ca^2+^ activated enzyme activity to near membrane microdomains within the cell.

## 2 The concept of the model

**Fig. 1:**
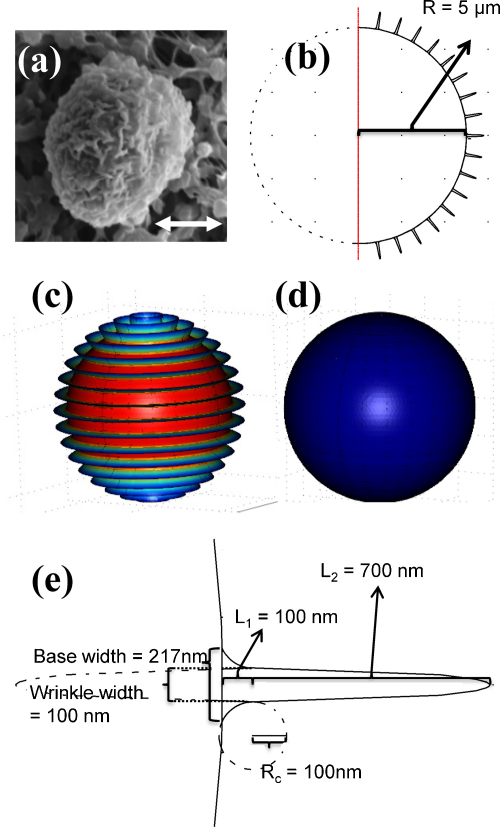
The wrinkled surface of neutrophils. The figure shows (a) the wrinkled structure of an unstimulated neutrophil imaged using scanning electron microscopy (scale bar = 5 μm). (b) The wrinkled surface segment generated in our model, based on a half circle with a radius of 5 μm. (c) Its rotation about the *z*-axis to give a 3D wrinkled sphere. (d) A similar method was adopted to generate the mathematically smooth sphere. (e) The wrinkle geometry was based on an ellipse where the major axis (*L*_2_, taken here as 1400 nm) and the minor axis (*L*_1_, taken here as 100 nm), is connected to the surface with two circles with a radius of 100 nm. The base of the wrinkle is more than twice as wide as the ellipse—in this case around 217 nm.

In order to generate a wrinkled cell surface to investigate the effect of Ca^2+^ influx, we created a 2D wrinkled surface segment (Fig. 1b) with intracellular node points from which Ca^2+^ concentration was calculated using finite element method. All simulations were made in 2D using axial symmetry and cylindrical coordinates. By rotation of this segment about its *z*-axis, the corresponding 3D surface was created which included parallel wrinkles (Fig. 1c). Although the wrinkles on the surfaces of actual cells are at random orientations (Fig. 1a), the model wrinkles have the same appropriate cross-section in 2D and are extended membrane folds as in the real-life situation (Fig. 1c). Furthermore, the surface area of the wrinkles matches that in real cells. The same algorithms were used to calculate cytosolic free Ca^2+^ changes in both the wrinkled surface and the smooth surfaced model (Fig. 1d). The Ca^2+^ concentration was calculated using standard equations for diffusion of free and buffered Ca^2+^, influx of Ca^2+^ and ATP driven Ca^2+^ extrusion. The model includes the three variables: cytosolic free Ca^2+^, Ca^2+^ bound to intracellular buffer and free Ca^2+^ buffer. The partial differential equations used to calculate changes in the concentration of free Ca^2+^, protein and protein bound Ca^2+^ are;

(1) $$\frac{\partial [\text{C}{\text{a}}^{2+}]}{\partial t}=D_{\text{C}{\text{a}}^{2+}}\nabla^{2}[\text{C}{\text{a}}^{2+}]-R$$

(2) $$\frac{\partial [\text{Buffer}]}{\partial t}=D_{\text{Buffer}}\nabla^{2}[\text{Buffer}]-R$$

(3) $$\frac{\partial [\text{C}{\text{a}}^{2+}:\text{Buffer}]}{\partial t}=D_{\text{C}{\text{a}}^{2+}:\text{Buffer}}\nabla^{2}[\text{C}{\text{a}}^{2+}:\text{Buffer}]+R$$

where *D*_*i*_ is diffusion constants and *R* is the reaction:

(4) $$R=k_{f}[\text{C}{\text{a}}^{2+}]\cdot [\text{Buffer}]-k_{r}[\text{C}{\text{a}}^{2+}:\text{Buffer}]$$

where *k*_*f*_ and *k*_*r*_ are the rate constants (see Table 1). At the central axis axial symmetry was used for all species. For Buffer and Ca^2+^ bound buffer (Ca^2+^:Buffer) the boundary at the membrane was modelled with symmetry/insulation. For cytosolic free Ca^2+^ the boundary condition at the membrane surface is given by the flux:

(5) $$J=P_{rest}({\left[\text{C}{\text{a}}^{2+}\right]}_{ext}-[\text{C}{\text{a}}^{2+}])-\frac{J_{efflux}[\text{C}{\text{a}}^{2+}]}{K_{m}+[\text{C}{\text{a}}^{2+}]}+k_{open}\times \frac{J_{stim}}{Area}$$

The first term in this expression represents the resting flux of Ca^2+^ across the membrane. The second term represents the pumping of Ca^2+^ out of the cell, while the third term represents the Ca^2+^-influx following stimulation of the cell. Although it is possible that some Ca^2+^ influx channels may be localized to surface projections such as sensory microvilli [24], we have taken the conservative assumption that Ca^2+^ channels were distributed equally over the cell membrane. In this way, the model had not an in-built bias towards higher cytosolic free Ca^2+^ within the wrinkled areas of membrane.

### 2.1 The details of the model

The parameters used in the equations and the initial conditions are listed in Tables 1 and 2, respectively. Using the same numerical values for Ca^2+^ influx, efflux, buffering and diffusion, we have modelled two cases; one of a smooth spherical cell surface, and the other for a more realistic topology with a “wrinkled” surface. The terms describing the flux of Ca^2+^ across the membrane is taken from neutrophils, because in these cells, surface wrinkles are of a particular interest and partly because many of the parameters required have been quantified in these cells. The model, however, employs the essential features of Ca^2+^ modelling from other models [2,25–29] and diffusion terms which are assumed to be general for all cells. The model can therefore be generalised to any cell type and the effect of surface wrinkling on the generation of high Ca^2+^ microdomains established.

### 2.2 Model parameters

#### 2.2.1 Ca^2+^ pumping

The passive Ca^2+^ leak across the plasma membrane (*P*_*rest*_([Ca^2+^]_*ext*_ − [Ca^2+^])) (Eq. (5)) is balanced by a pumped efflux across the membrane (*J*_*efflux*_[Ca^2+^]/(*K*_*m*_ + [Ca^2+^])) mediated by a Ca^2+^-ATPase (Eq. (5)) [30,31]. The maximal efflux of Ca^2+^ (*J*_*efflux*_) was therefore calculated to balance influx resultant from a resting permeability coefficient for Ca^2+^ of 8 cm/s [32–34]. Other pumping rates were calculated assuming Henri–Michaelis–Menten kinetics, which were qualitatively similar to results obtained if we instead used Hill kinetics [31]. The affinity of Ca^2+^ for the Ca^2+^-ATPase was set as constant *K*_*m*_ = 1.5 μM. Although the affinity may increase following calmodulin activation [31], the model was not qualitatively sensitive to changes in this parameter over the time scale of our simulations (0.1–5 s) (data not shown).

#### 2.2.2 Stimulated Ca^2+^ influx

**Fig. 2:**
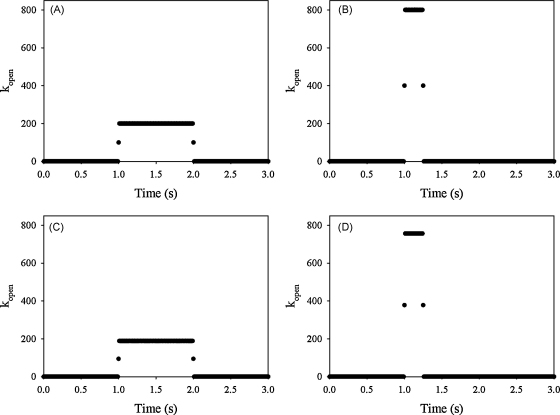
Activation of Ca^2+^ influx. The additional influx is modeled using the variable *k*_*open*_, that increases from 0 to a value over a short time and then again decreases to 0. When the additional Ca^2+^ influx is active for 1 s in the model with wrinkles (A) *k*_*open*_ has the value of 200 for 1 s and (C) in the model without wrinkles it has the value of 189 (also for 1 s). In the simulation where the influx of Ca^2+^ is active for 0.25 s *k*_*open*_ is 800 in the wrinkled model (B) and 756 in the model without wrinkles (D). Note that the area under the curves in (A) and (B) are the same and also in (C) and (D), so the total influx of Ca^2+^ due to additional activation is the same.

A number of different stimuli generate a large increase in cytosolic Ca^2+^ in neutrophils. There is evidence to suggest that part of the influx is mediated by the non-selective cationic TRP channels, especially TRPM2 [35,36]. We estimate that the number of open channels must be at least 100–150 cell^−1^ during the first second of stimulation for a Ca^2+^ influx sufficiently large to generate the observed change in bulk cytoplasmic Ca^2+^ concentration [36]. It is assumed that the opening of Ca^2+^ channels is randomly distributed on the cell surface in the same way as Ca^2+^-ATPase. The effect of channel opening on cytosolic free Ca^2+^ (*k*_*open*_) depends on the time at which there is an increased open probability, which is shown in Fig. 2 for 1 s (Fig. 2A) and for 0.25 s (Fig. 2B) when we simulate the influx in the wrinkled model.

When we simulate the Ca^2+^ influx in the model without wrinkles we use the functions shown in Fig. 2C and D which essentially are the same as Fig. 2A and B, but has a slightly lower value such that the change in the bulk concentration of Ca^2+^ is the same. The additional influx is modelled using a built-in continuous function to simulate a step function. The extra influx is modelled as if it is independent on the extracellular Ca^2+^ concentration and we have normalized the influx such that the total Ca^2+^ influx is the same in the smooth surface and the wrinkled surface model. This implies that the influx pr membrane area is higher in the model without wrinkles.

#### 2.2.3 Cytosolic Ca^2+^ buffering and diffusion

Cytosolic Ca^2+^ buffering results from binding of Ca^2+^ to both proteins and small molecules, which in neutrophils is equivalent to a total buffer concentration of 0.76 mM with an average *K*_*d*_ of 0.5 μM [37]. We model the buffering with an equilibrium reaction and model all three species (Eqs. (1)–(3)). Although the buffer is a mixture of a diverse group of molecules, we have used previously published diffusion constants for Ca^2+^ and the molecules that buffer Ca^2+^ [38].

#### 2.2.4 Geometry

The radius of the spherical surface of the neutrophil was taken as 5 μm (Fig. 1b), on which were superimposed wrinkles perpendicular to the membrane pointing away from the centre of the cell (Fig. 1e). The wrinkles are based on an ellipse which is 1400 nm long and 100 nm wide. The ellipse is connected to the “cell” 100 nm away from the cell using two circles with a radius of 100 nm. Thus, the wrinkles are 100 nm wide 100 nm from the cell surface and more than 200 nm wide at the surface (Fig. 1e). In a related myeloid cell type, similar wrinkles have been shown to have a width at the base of 100 nm and a height above the spherical surface of 800 nm [6]. In neutrophils scanning electron microscopy suggests that their surface wrinkles are essentially similar [39]. The model of the cell therefore has wrinkles perpendicular to the cell surface and pointing directly at the centre of the cell (Fig. 1b). By revolving the segment about its *z*-axis, a 3D surface is generated which has similarity to the wrinkled surface of a neutrophil. The same approach was used to generate the smooth cell model. The surface area of the wrinkled cell is 871 μm^2^, where 73% of the membrane is in the wrinkles. The smooth cell has a surface area of 314 μm^2^. The total length of the wrinkles is 446 μm as measured from where the wrinkle begins to protrude out of the cell membrane (see Fig. 1e). Approximately 4% of total cell volume is in wrinkles.

#### 2.2.5 Model implementation

The smooth and wrinkled models were implemented and solved in COMSOL Multiphysics, Chemical Engineering Module (COMSOL AB.). They were simulated using Direct (Pardiso) solver, with relative and absolute tolerances of 1E−7 and 1E−8, respectively, and with the time step restricted to maximum 0.1 s. The models are available as a COMSOL model report (supplementary material 1).

## 3 Results

### 3.1 Effect of Ca^2+^ influx on sub-plasma membrane Ca^2+^ concentration

**Fig. 3:**
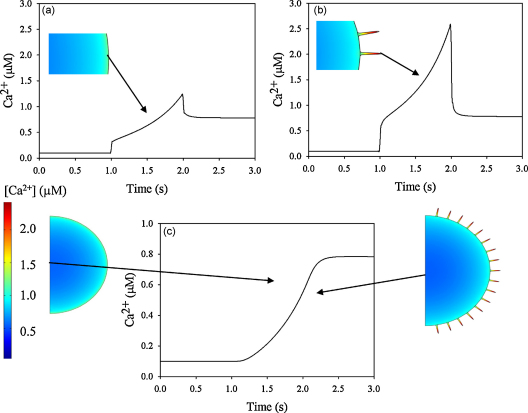
Simulations of stimulated Ca^2+^ influx (1 s duration). The near membrane Ca^2+^ concentration is shown for (a) the smooth cell model (5 nm beneath the plasma membrane as indicated with the arrow) and (b) the wrinkled cell model (5 nm from tip of wrinkle as indicated with the arrow at the red mark). The concentration of Ca^2+^ in the centre of either model cell is similar as shown in (c). The concentration of Ca^2+^ at time 2.0 s is visualised in the two models with colours using the same colour range as shown in (c). For these simulations, the effect of 1 s duration Ca^2+^ influx (1–2 s on time scale) is shown using the characteristics shown in Fig. 2A and B. (For interpretation of the references to color in this figure legend, the reader is referred to the web version of the article.)

As a high sub-plasma membrane Ca^2+^ concentration is proposed to be functionally important in both exocytsosis [21] and membrane unwrinkling during phagocytosis [10] and cell “spreading” [11] of neutrophils, we have in the simulations used parameters which are applicable to micro-anatomy and Ca^2+^ in these cells. The values and published sources of these parameters are given in Tables 1 and 2. The exact nature, number or distribution of Ca^2+^ influx channels on the surface of neutrophils is not known, but it can be estimated that there are at least 100–150 channels/cell (see above). Assuming a random distribution of the channels 70–100 of these are situated in the wrinkles. When simulated Ca^2+^ influx was run for a Ca^2+^ influx phase of 1 s period, the bulk cytosolic free Ca^2+^ rose after a delay of about 100 ms to a value of about 800 nM (Fig. 3c) in both the smooth and the wrinkled cell. The timing and extent of this rise agree with the timing and magnitude experimentally determined in neutrophil populations, suggesting that simple diffusion of influxing Ca^2+^ is the dominant mechanism for the bulk Ca^2+^ signal as in chick sensory neurones [40]. Similar relationships were found for Ca^2+^ influx occurring uniformly across either the smooth or the wrinkled spherical surface. However, Ca^2+^ concentrations 5 nm from the end of the horizontal wrinkle (i.e. parallel to the *z*-axis, as indicated in Fig. 3b), were significantly higher than 5 nm beneath the plasma membrane of the smooth sphere (Fig. 3a and b). In all subsequent analyses, we have similarly taken these two points as measures of “the near membrane Ca^2+^ concentration”. The initial rise in cytosolic Ca^2+^ occurred at the onset of Ca^2+^ channel opening, and was followed by a further rise during open channel period. In the simulation, this was followed by an abrupt decline to the bulk cytosolic level when the channels were closed (Fig. 3a and b).

### 3.2 Effect of Ca^2+^ buffer diffusion parameters on simulation

**Fig. 4:**
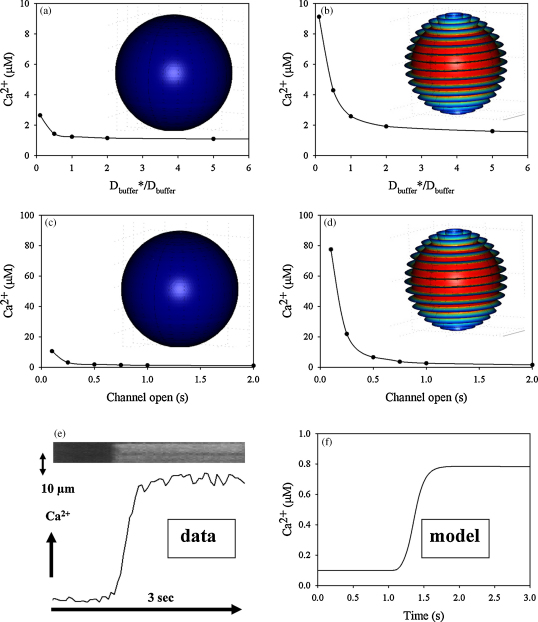
Effect of Ca^2+^ buffer diffusion and influx timing parameters of simulations of stimulated Ca^2+^ influx. Peak Ca^2+^ concentration, calculated as in Fig. 3, are shown (a and b) with different values of the protein diffusion constants (*D*_buffer_ and *D*_Ca:buffer_) and (c and d) with different channel open times. In (a and b) the channel open time is constant 1 s and in (c and d) the diffusion constants are the same as in Table 1. The ratio $D_{\text{buffer}}^{*}/D_{\text{buffer}}$ indicates the relative change of the diffusion constant for the buffer with respect to the diffusion constant in Table 1 (*D*_buffer_). Ca^2+^ influx periods in (a and c) the smooth cell model and (b and d) the wrinkled cell model. For different times of Ca^2+^ influx, the constant *k*_*open*_ was adjusted to give the same net change in global cytosolic Ca^2+^ (see Fig. 2). (e) An example of the global Ca^2+^ change in a single human neutrophil driven by Ca^2+^ influx, stimulated with f-met-leu-phe (1 μM), is shown as a confocal xt scan of a fluo3-loaded neutrophil and conventional time course. (f) This is compared with the model prediction for Ca^2+^ influx for 0.25 s.

The additional Ca^2+^ within the wrinkles arose in part because Ca^2+^ influx occurred across a larger surface area than for the equivalent sub-membrane region in the smooth model. However, as the larger surface area also included additional Ca^2+^ extrusion pumps, the extent of the Ca^2+^ rise will depend on the rate of diffusion of free and bound Ca^2+^ out of the narrow mouth of the wrinkle. As the values for Ca^2+^ diffusion have not been accurately determined in neutrophils, we used the published values for free and bound Ca^2+^ diffusion for oocyte cytosol as $D_{\text{C}{\text{a}}^{2+}}=233\;\mu {\text{m}}^{2}/\text{s}$; *D*_buffer_ = 13 μm^2^/s; $D_{\text{C}{\text{a}}^{2+}:\text{buffer}}=13\;\mu {\text{m}}^{2}/\text{s}$ [38]. While the diffusion of free Ca^2+^ is unlikely to differ significantly in different cells, the diffusion of “bound Ca^2+^” would depend on the nature of the cellular Ca^2+^ buffer. As neutrophils have a high cytosolic Ca^2+^ buffering capacity [37,41] we therefore investigated the effect of diffusion of the buffer on the model. In the smooth surface model, reduced diffusion of buffered Ca^2+^ would have little effect on the peak Ca^2+^ concentration (Fig. 4a). However, the peak Ca^2+^ concentration in the wrinkles is sensitive to this parameter and rises steeply as the diffusion constant of buffered Ca^2+^ is reduced (Fig. 4b). However, as this parameter cannot be measured locally in neutrophils, in subsequent simulations the “standard” diffusion parameters have been used.

### 3.3 Effect of Ca^2+^ influx parameters on simulation

The parameter which gives the largest effect in our model is the Ca^2+^ channel opening time (Fig. 4c and d). If this period is reduced, while the number of open channels is adjusted to give the same Ca^2+^ influx (see Fig. 2), there is little effect on the peak sub-membrane Ca^2+^ generated in the smooth surface model (Fig. 4c). In contrast, reducing the Ca^2+^ channel opening time to 0.25 s or 0.1 s, increases the peak Ca^2+^ in wrinkles to 20 μM and 80 μM, respectively (Fig. 4d). In our initial simulations, we took the Ca^2+^ rise time in neutrophil populations as an estimate of the timing of this increased Ca^2+^ channel open probability to about 1 s. However, the responses of neutrophils are asynchronous in the subsecond time scale, with individual cells having variable delays [42]. The population response is thus a time-averaged signal. When the rise of cytosolic free Ca^2+^ is monitored in individual neutrophils, the Ca^2+^ rise is actually faster, occurring over 100–250 ms (Fig. 4e). Similar kinetics to those observed experimentally are predicted by our simulations for the period of Ca^2+^ channel opening of around 250 ms (Fig. 4e and f). With an influx time of 0.25 s, the model predicts that cytosolic Ca^2+^ concentration at the centre of the cell will start to increase about 100 ms after the Ca^2+^ influx is initiated and that the plateau is reached 0.8 s after initiation of Ca^2+^ influx (Fig. 4f). The apparent time delay between the onset of Ca^2+^ influx and until the maximum concentration is reached in the cell is also within the same order as observed experimentally [43] (see Figs. 3 and 4 [43]).

### 3.4 Large intra-wrinkle Ca^2+^ concentration changes

**Fig. 5:**
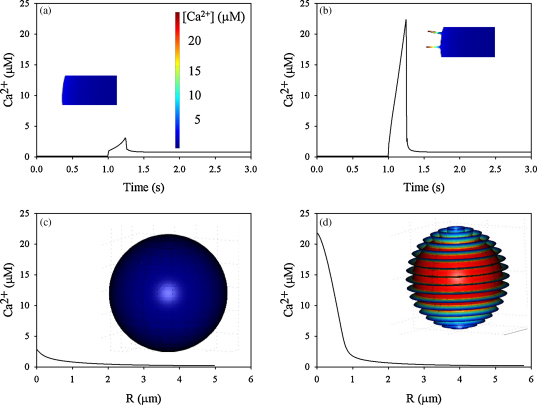
Intra-wrinkle Ca^2+^ concentration changes (0.25 s influx). The near membrane Ca^2+^ concentration is shown for (a) the smooth cell model (5 nm beneath the plasma membrane, same spot as in Fig. 3) and (b) the wrinkled cell model (5 nm from tip of wrinkle, same spot as in Fig. 3) when the additional Ca^2+^ flux is active between time 1 and 1.25 s. The concentration of Ca^2+^ at time 1.25 s is visualised in the two models with colours using the same colour range as shown in (a). The concentration of Ca^2+^ at time 1.25 s (peak concentration) presented as a cross-section through the cell is presented in (c and d), where the *y*-axis is the distance from the cell membrane (c) and the distance from the tip of the wrinkle (d), and in both situations the cross-section is made through the point where Ca^2+^ is measured in (a) and (b). For these simulations, the effect of 0.25 s duration Ca^2+^ influx (1–1.25 s on time scale) is shown using the characteristics shown in Fig. 4.

**Fig. 6:**
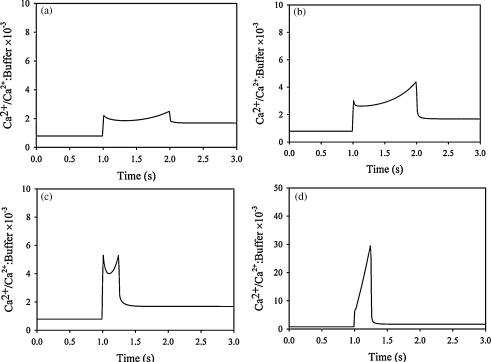
The ratio between free Ca^2+^ and bound Ca^2+^. The ratio between free and bound Ca^2+^ when the influx is active for 1 s (see Fig. 3) with the smooth (a) and the wrinkled geometry (b). When the influx is reduced to 0.25 s (see Fig. 5) the ratio in slightly increased in the model without wrinkles (c) and in the model with wrinkles more than 10-fold (d).

Using experimentally determined timing and magnitudes for the bulk Ca^2+^ signal, the model predicts significantly raised intra-wrinkle Ca^2+^ concentrations of near 20 μM (Fig. 5a and b, and movie 3 (supplementary material)) over a significant volume of cytosol (about 4% of the total cell volume as mentioned earlier). Under these conditions, the distribution of Ca^2+^ concentrations with respect to the distance to the cell surface within the cell at the time when Ca^2+^ peaked (0.25 s after the additional influx is activated), shows a clear boundary approximately 1 μm within the cell (Fig. 5c and d), where the cytosolic Ca^2+^ is essentially the same as predicted for the smooth surface. However, in the outer most micron, Ca^2+^ rises with a different gradient as the wrinkle is entered (Fig. 5c and d). The sudden rise in free Ca^2+^ (Fig. 5b) exists because the amount of free buffer is exhausted locally. The ratio between free Ca^2+^ and bound Ca^2+^ is plotted in Fig. 6. Before the influx is started the ratio is 7.9 × 10^−4^ close to membrane, as a result of the steady state between influx and efflux of Ca^2+^. The ratio changes less than a factor of 10 following Ca^2+^ influx. However, in the wrinkled surface model, the ratio changes by a factor of 30 during 0.25 s Ca^2+^ influx and the buffer is almost depleted locally (Fig. 6d). It should be noted that the total cellular buffer capacity is not exhausted (Fig. 6d), but that the effect is localised to the wrinkles. This is because free buffer from the bulk cytosol cannot diffuse into the wrinkles sufficiently fast to replace the Ca^2+^ bound buffer. This is in contrast to the smooth surface model, where diffusion of the free buffer is unrestricted (Fig. 6c). The general conditions required for establishing a local high Ca^2+^ domain can therefore be defined as a region of cytosol having rapid access to Ca^2+^ whose buffering capacity is limited or not easily refilled. In such cases, a microdomain of high Ca^2+^ may be generated. This outermost micron is thus a microdomain whose Ca^2+^ concentration rises to significantly higher levels than the bulk cytosolic concentration. This concentration of Ca^2+^ may well represent a lower limit since the diffusion constants of buffered Ca^2+^ in small cells may be lower than in the larger oocytes which we used here. The presence of organelles close to the membrane could add further restrictions to the diffusion and increase the magnitude of the microdomains.

**Fig. 7:**
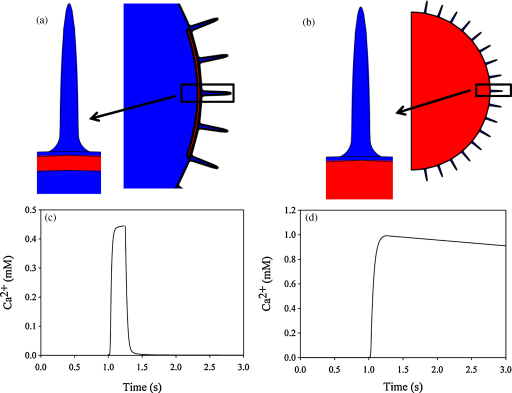
Organelles increase the magnitude of intra-wrinkle Ca^2+^ domains. Two scenarios are shown (a) endoplasmic reticulum is located 20 nm under the entire plasma membrane and covers the mouths of just 3 wrinkles and (b) endoplasmic reticulum is 20 nm under a part of the plasma membrane. In both cases, where red is the organelle and blue is the cytosol. The endoplasmic reticulum thickness was 10 nm thick in (a) but, for clarity, is shown thicker. The change in intra-wrinkle Ca^2+^ concentration measured 5 nm from the end of the tip of the wrinkle (c and d) for a Ca^2+^ influx pulse of 0.25 s (between 1 s and 1.25 s) across the membrane of the geometry shown in the corresponding panel above. The constant *J*_*stim*_ is multiplied with (1 − [Ca^2+^]/[Ca^2+^]_*ext*_) in these simulations and the uptake of Ca^2+^ into the endoplasmic reticulum is modelled as the transport of Ca^2+^ across the plasma membrane (*J*_*efflux*_ [Ca^2+^]/(*K*_*m*_ + [Ca^2+^]).

Since Ca^2+^ storage organelles, endoplasmic reticulum and sarcoplasmic reticulum, can be located within 25 nm of the plasma membrane [44,45], and create Ca^2+^ microdomains [46,47], it was important to investigate whether such located organelles influence the topology generated Ca^2+^ microdomains. Assuming that these organelles take-up Ca^2+^ with standard kinetics (*J*_*efflux*_ [Ca^2+^]/(*K*_*m*_ + [Ca^2+^]) and with unlimited capacity and with no leakage, Ca^2+^ microdomains within the wrinkles would be elevated further. To compensate for near extracellular concentrations of Ca^2+^ in this model we have scaled the influx parameter *J*_*stim*_ with (1 − [Ca^2+^]/[Ca^2+^]_*ext*_) in all the simulations. If only a part of the membrane was covered the near membrane Ca^2+^ concentration in the wrinkles increased to around 0.45 mM (Fig. 7a and c), or 1 mM when the entire membrane was sheltered (Fig. 7b and d). These effects are due to the organelle near the wrinkle obstructing diffusion of the buffer, bound Ca^2+^ and free Ca^2+^ out of the wrinkle. If the dimension of the uptake organelle covering a few wrinkles was wider than 0.8 μm near millimolar Ca^2+^ domains remained. Increasing the influx of Ca^2+^ into the organelle by a factor of 10 or 100 also had only little impact on the Ca^2+^ microdomains. To reduce the Ca^2+^ domains to 25 μM, the maximum uptake into the organelles must be increased by more than a factor of 1000 or the Ca^2+^ influx reduced to 13.5% of that used previously. When the influx of Ca^2+^ is just 20% of that in Fig. 5b, the model predicts the existence of Ca^2+^ microdomains with a concentration of 0.1 mM. In both cases the global Ca^2+^ concentration was only changed by less than 45 nM. These simulations show that organelles if very close to the wrinkles will increase the magnitude of the Ca^2+^ microdomains. It should be noted that in a number of cell types, including neutrophils, the endoplasmic reticulum does not extend to the plasma membrane, and can thus have only little influence on the intra-wrinkle Ca^2+^ concentration.

### 3.5 Effect of wrinkle topology on Ca^2+^ microdomains

In this study, we have modelled the cell surface topology using the winkle dimensions reported by scanning electron microscopy [6]. However, although these dimensions provide a good estimate of mean wrinkle structure, wrinkles may exist in a range of sizes. For example, under transmission electron microscopy, the wrinkles appear as “microvilli” with lengths of 50–1900 nm and base widths of 150–200 nm [48,49] and biophysical measurement suggest the functional lengths of the wrinkles to be only 300 nm [49]. It is therefore important to establish what influence the wrinkle dimensions have on the intra-wrinkle Ca^2+^ concentration.

We have therefore repeated the modelling study using the extremes dimensions for the wrinkles but maintaining the overall geometry whereby approximately 70% of the membrane is localised in the wrinkles in accordance with reported estimates [6,48,49].

**Fig. 8:**
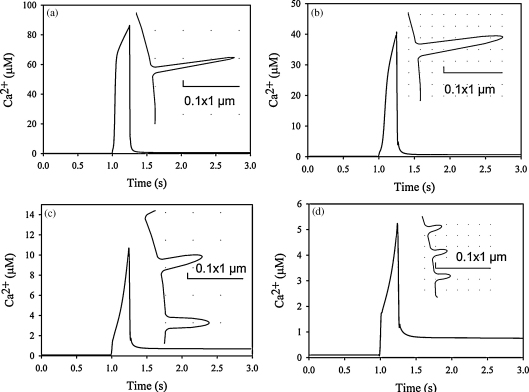
The Ca^2+^ domain depends on the structure of the wrinkle. The intra-wrinkle Ca^2+^ concentration (5 nm from the end of the wrinkle tip) is shown in wrinkled models where the geometries have been changed. In these examples, the wrinkles were (a) 1500 nm long (Fig. 1, *L*_1_ = 100 nm, *L*_2_ = 1400 nm) and 100 nm wide (b) 1500 nm long (Fig. 1, *L*_1_ = 100 nm, *L*_2_ = 1400 nm) and 200 nm wide (c) 800 nm (Fig. 1, *L*_1_ = 100 nm, *L*_2_ = 700 nm) and 200 nm wide and (d) 300 nm (Fig. 1, *L*_1_ = 100 nm, *L*_2_ = 200 nm) and 100 nm wide. In each graph, the insert shows the geometry of a wrinkle and the size is illustrated with the bars that are 100 nm × 1000 nm.

Within surface wrinkles which were 1500 nm long (Fig. 1e, *L*_1_ + *L*_2_ = 1500 nm) and 100 nm wide (base width), Ca^2+^ concentrations were even higher than in our previous model, reaching around 85 μM (Fig. 8a). When the width of these wrinkles was increased to 200 nm, the intra-wrinkle Ca^2+^ concentration was reduced but remained high at 40 μM (Fig. 8b). A similar lowering of intra-wrinkle Ca^2+^ was observed when the standard length (800 nm) wrinkles were widened to 200 nm, intra-wrinkle Ca^2+^ peaking at around 11 μM (Fig. 8c). Conversely, narrowing the wrinkle width to 50 nm elevated intra-wrinkle Ca^2+^ concentrations to 45 μM.

Not surprisingly, as the wrinkle length is reduced, the surface of the cell approximates more closely to the smooth sphere. However, even within wrinkles just 300 nm long (Fig. 1e, *L*_1_ + *L*_2_ = 300 nm) and 100 nm base width, the Ca^2+^ concentration is elevated at 5.2 μM (Fig. 8d). As before, increasing the base width of these wrinkles reduces the peak intra-wrinkle Ca^2+^. However, even with a short stubby wrinkle 300 nm long and 200 nm wide, the Ca^2+^ concentration within is higher than the bulk cytosol by an additional 1 μM.

It was concluded that high Ca^2+^ microdomains will be generated most effectively by the longer and more narrow membrane wrinkles, but that wrinkles of similar dimensions to those found experimentally can generate localised Ca^2+^ regions of nearly 0.1 mM.

## 4 Discussion

The experimental evidence that Ca^2+^ is extremely high in the cytosol within wrinkles is difficult to obtain, as the wrinkles themselves are sub-light microscopical objects. In dendritic spines of neurones, which are small anatomical structures, high Ca^2+^ can be observed during Ca^2+^ signalling [50]. These structures are more complex than simple cell wrinkles, having a “firewall” of ER at the base, which can release and take-up Ca^2+^. The underlying mechanisms for generating localised elevated intra-spine Ca^2+^ may therefore be different [51]. However, in the simple wrinkled membrane of neutrophils, there is evidence for the functional existence of a high Ca^2+^ sub-plasma membrane domains [10,11,21] and near membrane Ca^2+^ reported by a membrane associated Ca^2+^ indicator, FFP-18, is over 30 μM [52]. It should be noted that at non-wrinkled regions of tight neutrophil adherence to a solid substrate, at which total internal reflection fluorescence microscope measurement of Ca^2+^ can be made within 100 nm of the plasma membrane, Ca^2+^ peaks at only 1 μM [43]. This experimentally determined difference between near membrane Ca^2+^ concentrations at wrinkled and non-wrinkled neutrophil surface is predicted by our model.

The high sub-membrane Ca^2+^ concentration within the surface wrinkles are sufficient to activate proteins with *K*_*d*_'s of tens of micromolar Ca^2+^, such as calpain-1 [15–17], TRPM2 [22,23] and some isoforms of protein kinase C [53–55]. As all these examples are proteins located at the plasma membrane or associated with the cortical cytoskeleton which holds the wrinkles in place, they are strategically placed for activation within the wrinkled membrane. Although we have used the topology of the neutrophil as an example of the wrinkled surface, Bergmann glial cells which are far more convoluted, having a surface-to-volume ratio 13 times higher than neutrophils [56] also have microdomains of high Ca^2+^ which are found in their membrane projections [57]. Like neutrophils the endothelial cells also have microvilli [58], and Ca^2+^ microdomains have recently been detected in endothelial cells following influx of Ca^2+^ [59], and we conjecture that they could be generated due to the wrinkles. The microvilli of Drosophila photoreceptors also generate microdomains of high (20–200 μM) Ca^2+^ following light stimulation [60], which is crucial for the function of the receptor [61]. It thus seems likely that surface topology is important in a number of cell types for directing Ca^2+^ signalling to specific proteins with the Ca^2+^ microdomain it generates. As the model we present is simple both in geometry and biological assumptions, this raises the possibility that any cell with a non-smooth surface topology may exploit localised Ca^2+^ signalling within the wrinkled surface.

Although the Gouy–Chapman–Stern theory also provides explanation for elevated Ca^2+^ near the plasma membrane, it is applicable only within 2 nm of the membrane. This effect has been suggested in part to explain why PKC is activated at a bulk concentration of 700 nM Ca^2+^ [53]. We have not included the effect of the electrical double layer in the current model, but Gouy–Chapman theory [62] predicts the electrical surface potential (*ψ*_0_) as

$$2{C^{\prime \prime }}_{b}cosh^{2}\left(\frac{F\psi_{0}}{RT}\right)+{C^{\prime }}_{b}\cosh\left(\frac{F\psi_{0}}{RT}\right)-\left(2{C^{\prime \prime }}_{b}+{C^{\prime \prime }}_{b}+\frac{1}{2}{\left(\frac{\sigma }{A}\right)}^{2}\right)=0$$

$$A=\sqrt{2RT\varepsilon_{r}\varepsilon_{0}}$$

where ${C^{\prime \prime }}_{b}$ is the concentration of the divalent electrolyte, ${C^{\prime }}_{b}$ is the concentration of the monovalent electrolyte, *σ* is the electrical surface charge density, *R* is the gas constant, *T* is the absolute temperature and *ɛ*_0_ and *ɛ*_*r*_ are the absolute and relative permittivities, respectively.

Setting the concentration of monovalent cations $\left({C^{\prime }}_{b}\right)$ to 100 mM and divalent cations $\left({C^{\prime \prime }}_{b}\right)$ to 5 mM and assuming that the surface charge density *σ* is between −0.02 C/m^2^ and −0.05 C/m^2^ the *ψ*_0_ was calculated. If the surface charge density is small, the potential will decay exponentially with distance from the membrane surface with the coefficient *κ*:

$$\kappa =\sqrt{\frac{2Z^{2}F^{2}C_{b}}{RT\varepsilon_{0}\varepsilon_{r}}}$$

At the zeta potential where *ψ*_0_ equals *κ* (around 1 nm from the surface of the membrane) the Ca^2+^ concentration will be increased by a factor of 2–4 with respect to the bulk concentration. Thus, the effect of the electrical surface potential is far weaker than that due to surface topology and not sufficient to explain the presence of the high Ca^2+^ concentrations. However, the electrical surface potential will increase the concentration of cations near the membrane and hence may work as a local cation buffer. If we assume that the surface charge density is −0.02 C/m^2^, then the membrane can at most bind 1.8 × 10^−16^ mole of positive charges or 9 × 10^−17^ mole of Ca^2+^ if we neglect the presence of Mg^2+^ and assume that no other cations interfere with the membrane. The intracellular buffer can, on the other hand, bind 4 × 10^−16^ mole Ca^2+^. Therefore, the mobile buffer still remains the dominant buffer, which binds at least 4 times more Ca^2+^ than the membrane.

The wrinkles in this model are symmetrical structures and per se artificial, however, the real wrinkles also span the entire membrane and are connected in an almost similar geometric fashion (Fig. 1). Different approaches have been used to estimate the structure and dimension of the wrinkles [6,48,49]. All the estimates indicate that the width is between 100 nm and 200 nm, but caution must be taken with the estimates based on transmission electron microscopy as the wrinkles are 3D structures that are spanning the membrane and in case the section is not perpendicular to the direction of the wrinkle the width will be overestimated. In the model we present in Fig. 1 the wrinkles are 100 nm wide, 100 nm from the surface, and that is based on results obtained using scanning electron microscopy [6]. Using transmission electron microscopy it has been reported that the wrinkles might be wider [48,49], and we found that doubling the width decreased the near membrane Ca^2+^ concentration slightly (Fig. 8b). If the length of the wrinkles is decreased the topology will approach that of a smooth cell and hence the micro-environment provided by the wrinkles will disappear. On the other hand physics also sets an upper limit because if the wrinkles are too long they will break due to shear stress. To become activated the μ-calpains must bind Ca^2+^ and their *K*_*d*_ is between 10 μM and 50 μM [63,64]. The concentrations of Ca^2+^ obtained with wrinkles that are 800 nm long (Fig. 5) can easily explain the activation of μ-calpains near the membrane and even in wrinkles only 300 nm long there would also be a transient μ-calpain activity. When we extended the length of the wrinkles we found that the concentration of Ca^2+^ became close to 0.1 mM (Fig. 8), which is far more than needed to activate, e.g. the μ-calpains. Though the numbers for the length of the wrinkles vary between 300 nm and 1900 nm, the width is reported to be in a narrower interval from 100 nm to 200 nm. The wrinkles that are 800 nm long and 100 nm wide as described in Fig. 1 sets the ideal conditions for creating Ca^2+^ domains in the micromolar range that can activate proteins which otherwise would show little if any activity in the cytosol. If the structures were much wider or a lot shorter the apparent wrinkle structure would be lost and Ca^2+^ domains would disappear.

We simulated the Ca^2+^ influx with a deterministic approach, which is based on the assumption that the individual behaviour of different molecules can be neglected due to the number of the species according to the law of large numbers. One result of the simulations is that it is very likely that there are at least 800 active channels in the plasma membrane, which corresponds to 0.9 channel per μm^2^. With the assumption of a homogeneous distribution of the channels this implies that there are at least 584 channels in the wrinkles or 1.3 channels per μm wrinkle. The wrinkles have a total volume of 0.219 fl, which corresponds to about 4% of the total cell volume. A concentration of Ca^2+^ ions of 100 nM corresponds to about 1300 free Ca^2+^ ions in the wrinkles and when the concentration is 25 μM there are 3.3 × 10^5^ free Ca^2+^ ions in the wrinkles. Whether a system should be described as stochastic or deterministic depends on both the number of particles and the properties of the system as such. The number of free Ca^2+^ ions in the wrinkles following activation is around the deterministic limit as reported by Kummer et al. [65]. The Ca^2+^ dynamics inside the wrinkles in single neutrophils have not yet been measured, but global cytosolic Ca^2+^ measurements of neutrophils suggests that there is a stochastic element [55].

It is important to stress that the microdomains of high Ca^2+^ predicted by our simulations are not generated by assuming non-uniform distributions of Ca^2+^ channels, pumps, or Ca^2+^ release sites or by proposing new molecular properties for Ca^2+^ channels. The zones of high Ca^2+^ arise simply by including the known micro-anatomy of cell surfaces in the simulation. The work we report here has therefore highlighted the importance of including membrane surface topology when considering a model of chemical behaviour in cells.

## Figures and Tables

**Table 1:** List of constants.

Constant	Value	References
*P*_*rest*_	8 × 10^−5^ μm/(s cell)	[32–34]
[Ca^2+^]_*ext*_	1 mM	
*J*_*efflux*_	1.28 × 10^−15^ μmol/(μm^2^ s)	Estimated
*K*_*m*_	1.5 μM	[31]
*J*_*stim*_	9.3 × 10^−13^ μmol/(s cell)	Estimated
*Area* (wrinkled model)	8.706643 × 10^2^ μm^2^	
*Area* (smooth)	3.122003 × 10^2^ μm^2^	
*k*_*f*_	50 × 10^6^ μM/s	[2,25–29]
*k*_*r*_	25 l/s	[2,25–29]
$D_{\text{C}{\text{a}}^{2+}}$	233 μm^2^/s	[38]
*D*_Buffer_	13 μm^2^/s	[38]
*D*_Ca:Buffer_	13 μm^2^/s	[38]

**Table 2:** Initial conditions for variables.

Variable	Value	References
[Ca^2+^]_*cyt*_	100 nM	[13]
[Buffer]	0.633 mM	[37]
[Ca:Buffer]	0.126 mM	[37]

## References

[1] Llinas R., Sugimori M., Silver R.B. (1995). The concept of calcium concentration microdomains in synaptic transmission. Neuropharmacology.

[2] Naraghi M., Neher E. (1997). Linearized buffered Ca^2+^ diffusion in microdomains and its implications for calculation of [Ca^2+^] at the mouth of a calcium channel. J. Neurosci..

[3] Chad J.E., Eckert R. (1984). Calcium domains associated with individual channels can account for anomalous voltage relations of CA-dependent responses. Biophys. J..

[4] Zhou Y., Doerschuk C.M., Anderson J.M., Marchant R.E. (2004). Biomaterial surface-dependent neutrophil mobility. J. Biomed. Mater. Res. A.

[5] Bessis M. (1973). Living Blood Cells and Their Ultrastructure.

[6] Burwen S.J., Satir B.H. (1977). Plasma membrane folds on the mast cell surface and their relationship to secretory activity. J. Cell Biol..

[7] Erlandsen S.L., Hasslen S.R., Nelson R.D. (1993). Detection and spatial distribution of the beta 2 integrin (Mac-1) and l-selectin (LECAM-1) adherence receptors on human neutrophils by high-resolution field emission SEM. J. Histochem. Cytochem..

[8] Tohya K., Kimura M. (1998). Ultrastructural evidence of distinctive behavior of l-selectin and LFA-1 (alphaLbeta2 integrin) on lymphocytes adhering to the endothelial surface of high endothelial venules in peripheral lymph nodes. Histochem. Cell Biol..

[9] Petty H.R., Hafeman D.G., McConnell H.M. (1981). Disappearance of macrophage surface folds after antibody-dependent phagocytosis. J. Cell Biol..

[10] Hallett M.B., Dewitt S. (2007). Ironing out the wrinkles of neutrophil phagocytosis. Trends Cell Biol..

[11] Dewitt S., Hallett M. (2007). Leukocyte membrane “expansion”: a central mechanism for leukocyte extravasation. J. Leukoc. Biol..

[12] Kruskal B.A., Shak S., Maxfield F.R. (1986). Spreading of human neutrophils is immediately preceded by a large increase in cytoplasmic free calcium. Proc. Natl. Acad. Sci. U.S.A..

[13] Dewitt S., Hallett M.B. (2002). Cytosolic free Ca(2+) changes and calpain activation are required for beta integrin-accelerated phagocytosis by human neutrophils. J. Cell Biol..

[14] Sampath R., Gallagher P.J., Pavalko F.M. (1998). Cytoskeletal interactions with the leukocyte integrin beta2 cytoplasmic tail. Activation-dependent regulation of associations with talin and alpha-actinin. J. Biol. Chem..

[15] Goll D.E., Thompson V.F., Li H., Wei W., Cong J. (2003). The calpain system. Physiol. Rev..

[16] Wells A., Huttenlocher A., Lauffenburger D.A. (2005). Calpain proteases in cell adhesion and motility. Int. Rev. Cytol..

[17] Lebart M.C., Benyamin Y. (2006). Calpain involvement in the remodeling of cytoskeletal anchorage complexes. FEBS J..

[18] Bootman M.D., Berridge M.J., Lipp P. (1997). Cooking with calcium: the recipes for composing global signals from elementary events. Cell.

[19] Parker I., Choi J., Yao Y. (1996). Elementary events of InsP3-induced Ca^2+^ liberation in Xenopus oocytes: hot spots, puffs and blips. Cell Calcium.

[20] Hillson E.J., Hallett M.B. (2007). Localised and rapid Ca^2+^ micro-events in human neutrophils: conventional Ca^2+^ puffs and global waves without peripheral-restriction or wave cycling. Cell Calcium.

[21] Nusse O., Lindau M. (1988). The dynamics of exocytosis in human neutrophils. J. Cell Biol..

[22] Du J., Xie J., Yue L. (2009). Intracellular calcium activates TRPM2 and its alternative spliced isoforms. Proc. Natl. Acad. Sci. U.S.A..

[23] Csanady L., Torocsik B. (2009). Four Ca^2+^ ions activate TRPM2 channels by binding in deep crevices near the pore but intracellularly of the gate. J. Gen. Physiol..

[24] Liman E.R., Corey D.P., Dulac C. (1999). TRP2: a candidate transduction channel for mammalian pheromone sensory signaling. Proc. Natl. Acad. Sci. U.S.A..

[25] Wagner J., Keizer J. (1994). Effects of rapid buffers on Ca^2+^ diffusion and Ca^2+^ oscillations. Biophys. J..

[26] Nowycky M.C., Pinter M.J. (1993). Time courses of calcium and calcium-bound buffers following calcium influx in a model cell. Biophys. J..

[27] Marengo F.D., Monck J.R. (2000). Development and dissipation of Ca(2+) gradients in adrenal chromaffin cells. Biophys. J..

[28] Volfovsky N., Parnas H., Segal M., Korkotian E. (1999). Geometry of dendritic spines affects calcium dynamics in hippocampal neurons: theory and experiments. J. Neurophysiol..

[29] Holcman D., Schuss Z., Korkotian E. (2004). Calcium dynamics in dendritic spines and spine motility. Biophys. J..

[30] Lagast H., Pozzan T., Waldvogel F.A., Lew P.D. (1984). Phorbol myristate acetate stimulates ATP-dependent calcium transport by the plasma membrane of neutrophils. J. Clin. Invest..

[31] Carafoli E. (1991). Calcium pump of the plasma membrane. Physiol. Rev..

[32] Naccache P.H., Showell H.J., Becker E.L., Sha’afi R.I. (1977). Changes in ionic movements across rabbit polymorphonuclear leukocyte membranes during lysosomal enzyme release. Possible ionic basis for lysosomal enzyme release. J. Cell Biol..

[33] Tintinger G.R., Theron A.J., Steel H.C., Anderson R. (2001). Accelerated calcium influx and hyperactivation of neutrophils in chronic granulomatous disease. Clin. Exp. Immunol..

[34] Hallett M.B., Lloyds D. (1997). The Molecular and Ionic Signaling of Neutrophils.

[35] Wehage E., Eisfeld J., Heiner I., Jungling E., Zitt C., Luckhoff A. (2002). Activation of the cation channel long transient receptor potential channel 2 (LTRPC2) by hydrogen peroxide. A splice variant reveals a mode of activation independent of ADP-ribose. J. Biol. Chem..

[36] Heiner I., Eisfeld J., Warnstedt M., Radukina N., Jungling E., Luckhoff A. (2006). Endogenous ADP-ribose enables calcium-regulated cation currents through TRPM2 channels in neutrophil granulocytes. Biochem. J..

[37] von Tscharner V., Deranleau D.A., Baggiolini M. (1986). Calcium fluxes and calcium buffering in human neutrophils. J. Biol. Chem..

[38] Allbritton N.L., Meyer T., Stryer L. (1992). Range of messenger action of calcium ion and inositol 1,4,5-trisphosphate. Science.

[39] Hallett M.B., von Ruhland C.J., Dewitt S. (2008). Chemotaxis and the cell surface-area problem. Nat. Rev. Mol. Cell Biol..

[40] Coatesworth W., Bolsover S. (2008). Calcium signal transmission in chick sensory neurones is diffusion based. Cell Calcium.

[41] Al-Mohanna F.A., Hallett M.B. (1988). The use of fura-2 to determine the relationship between cytoplasmic free Ca^2+^ and oxidase activation in rat neutrophils. Cell Calcium.

[42] Pettit E.J., Hallett M.B. (1995). Early Ca^2+^ signalling events in neutrophils detected by rapid confocal laser scanning. Biochem. J..

[43] Omann G.M., Axelrod D. (1996). Membrane-proximal calcium transients in stimulated neutrophils detected by total internal reflection fluorescence. Biophys. J..

[44] Wellman G.C., Nelson M.T. (2003). Signaling between SR and plasmalemma in smooth muscle: sparks and the activation of Ca^2+^-sensitive ion channels. Cell Calcium.

[45] Vig M., Kinet J.P. (2009). Calcium signaling in immune cells. Nat. Immunol..

[46] Lederer W.J., Niggli E., Hadley R.W. (1990). Sodium-calcium exchange in excitable cells: fuzzy space. Science.

[47] Jafri M.S., Rice J.J., Winslow R.L. (1998). Cardiac Ca^2+^ dynamics: the roles of ryanodine receptor adaptation and sarcoplasmic reticulum load. Biophys. J..

[48] Bruehl R.E., Springer T.A., Bainton D.F. (1996). Quantitation of l-selectin distribution on human leukocyte microvilli by immunogold labeling and electron microscopy. J. Histochem. Cytochem..

[49] Shao J.Y., Ting-Beall H.P., Hochmuth R.M. (1998). Static and dynamic lengths of neutrophil microvilli. Proc. Natl. Acad. Sci. U.S.A..

[50] Petrozzino J.J., Pozzo Miller L.D., Connor J.A. (1995). Micromolar Ca^2+^ transients in dendritic spines of hippocampal pyramidal neurons in brain slice. Neuron.

[51] Finch E.A., Augustine G.J. (1998). Local calcium signalling by inositol-1,4,5-trisphosphate in Purkinje cell dendrites. Nature.

[52] Davies E.V., Hallett M.B. (1996). Near membrane Ca^2+^ changes resulting from store release in neutrophils: detection by FFP-18. Cell Calcium.

[53] Mosior M., Epand R.M. (1994). Characterization of the calcium-binding site that regulates association of protein kinase C with phospholipid bilayers. J. Biol. Chem..

[54] Fernandez-Chacon R., Konigstorfer A., Gerber S.H., Garcia J., Matos M.F., Stevens C.F., Brose N., Rizo J., Rosenmund C., Sudhof T.C. (2001). Synaptotagmin I functions as a calcium regulator of release probability. Nature.

[55] Newton A.C., Johnson J.E. (1998). Protein kinase C: a paradigm for regulation of protein function by two membrane-targeting modules. Biochim. Biophys. Acta.

[56] Reichenbach A., Siegel A., Senitz D., Smith T.G. (1992). A comparative fractal analysis of various mammalian astroglial cell types. Neuroimage.

[57] Grosche J., Matyash V., Moller T., Verkhratsky A., Reichenbach A., Kettenmann H. (1999). Microdomains for neuron-glia interaction: parallel fiber signaling to Bergmann glial cells. Nat. Neurosci..

[58] Middleton J., Patterson A.M., Gardner L., Schmutz C., Ashton B.A. (2002). Leukocyte extravasation: chemokine transport and presentation by the endothelium. Blood.

[59] Tomatis C., Fiorio Pla A., Munaron L. (2007). Cytosolic calcium microdomains by arachidonic acid and nitric oxide in endothelial cells. Cell Calcium.

[60] Wang T., Montell C. (2007). Phototransduction and retinal degeneration in Drosophila. Pflug. Arch..

[61] Gu Y., Oberwinkler J., Postma M., Hardie R.C. (2005). Mechanisms of light adaptation in Drosophila photoreceptors. Curr. Biol..

[62] Barber J. (1980). Membrane surface charges and potentials in relation to photosynthesis. Biochim. Biophys. Acta.

[63] Legendre J.L., Jones H.P. (1988). Purification and characterization of calpain from human polymorphonuclear leukocytes. Inflammation.

[64] Pontremoli S., Melloni E., Michetti M., Salamino F., Sparatore B., Horecker B.L. (1988). An endogenous activator of the Ca^2+^-dependent proteinase of human neutrophils that increases its affinity for Ca^2+^. Proc. Natl. Acad. Sci. U.S.A..

[65] Kummer U., Krajnc B., Pahle J., Green A.K., Dixon C.J., Marhl M. (2005). Transition from stochastic to deterministic behavior in calcium oscillations. Biophys. J..

