# Supplementary material for: Cell surface topology creates high Ca2+ signalling microdomains
Source: Cell Calcium. 2010 Apr;47(4):339–49. doi: 10.1016/j.ceca.2010.01.005 (PMC2877796; doi:10.1016/j.ceca.2010.01.005)
Supplement: Supplementary file 1 [file mmc1.doc]

**Model reports.**

Model reports generated by Comsol.

**Animation: Influx of Ca^2+^**

The Ca^2+^ concentration in the model with and without wrinkles is visualized during the additional Ca^2+^ influx. In (movie1.avi) and (movie2.avi) Ca^2+^ is shown using a colour code where blue corresponds to 0 mM and red to 2.5 µM Ca^2+^ as indicated by the colour bar. Between time 1 and 2 the additional influx is active. These animations correspond to Fig. 3 a and b respectively. In (movie3.avi) and (movie4.avi) Ca^2+^ is shown using a colour code where blue corresponds to 0 mM and red to 25 µM Ca^2+^ as indicated by the colour bar. Between time 1 and 1.25 the additional influx is active. These animations correspond to Fig. 5 a and b respectively. Because the colour codes span a large interval the changes will only be visible close to the membrane and in the wrinkles in accordance with Fig. 3 and 5.
