# Supplementary material for: Cell surface topology creates high Ca2+ signalling microdomains
Source: Cell Calcium. 2010 Apr;47(4):339–49. doi: 10.1016/j.ceca.2010.01.005 (PMC2877796; doi:10.1016/j.ceca.2010.01.005)
Supplement: Supplementary file 2 [file mmc2.pdf]

COMSOL Model Report

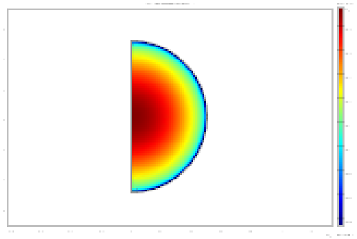

1. Table of Contents

- Title - COMSOL Model Report
- Table of Contents
- Model Properties
- Constants
- Global Expressions
- Geometry
- Geom1
- Extrusion Coupling Variables
- Solver Settings
- Postprocessing
- Equations
- Variables

2. Model Properties

Property	Value

Model name	
Author	
Company	
Department	
Reference	
URL	
Saved date	Mar 12, 2009 2:24:06 PM
Creation date	Dec 2, 2008 10:12:36 AM
COMSOL version	COMSOL 3.5.0.494

File name: C:\20090312-no_wrinkles.mph

Application modes and modules used in this model:

- Geom1 (Axial symmetry (2D))
  - Diffusion (Chemical Engineering Module)

3. Constants

Name	Expression	Value	Description
D_Ca	233[um^2/s]	(2.33e-10)[m^2/s]	
D_buffer	13[um^2/s]	(1.3e-11)[m^2/s]	
D_Cabound	13[um^2/s]	(1.3e-11)[m^2/s]	
start	1[s]	1[s]	
stop	2[s]	2[s]	
steep	.0001	1e-4	
Pstim	9.3e-19[mol/(s)]	(9.3e-19)[mol/s]	
area	3.122003e-10 [m^2]	(3.122003e-10)[m^2]	
Prest	8e-9[cm/(s)]	(8e-11)[m/s]	
Jefflux	1.28e-9 [mol/m^2/s]	(1.28e-9)[mol/($m^2 \cdot$ s)]	
CaExt	1[mmol/liter]	1[mol/m^3]	
Km	1.5[umol/liter]	0.0015[mol/m^3]	

kf	(50/1e-6)[1/((mol/liter)*s)]	50000[m^3/(s·mol)]	
kr	25[1/s]	25[1/s]	

4. Global Expressions

Name	Expression	Unit	Description
k_open	189*(flc2hs(t-start,steep)-flc2hs(t-stop,steep))		

5. Geometry

Number of geometries: 1

5.1. Geom1

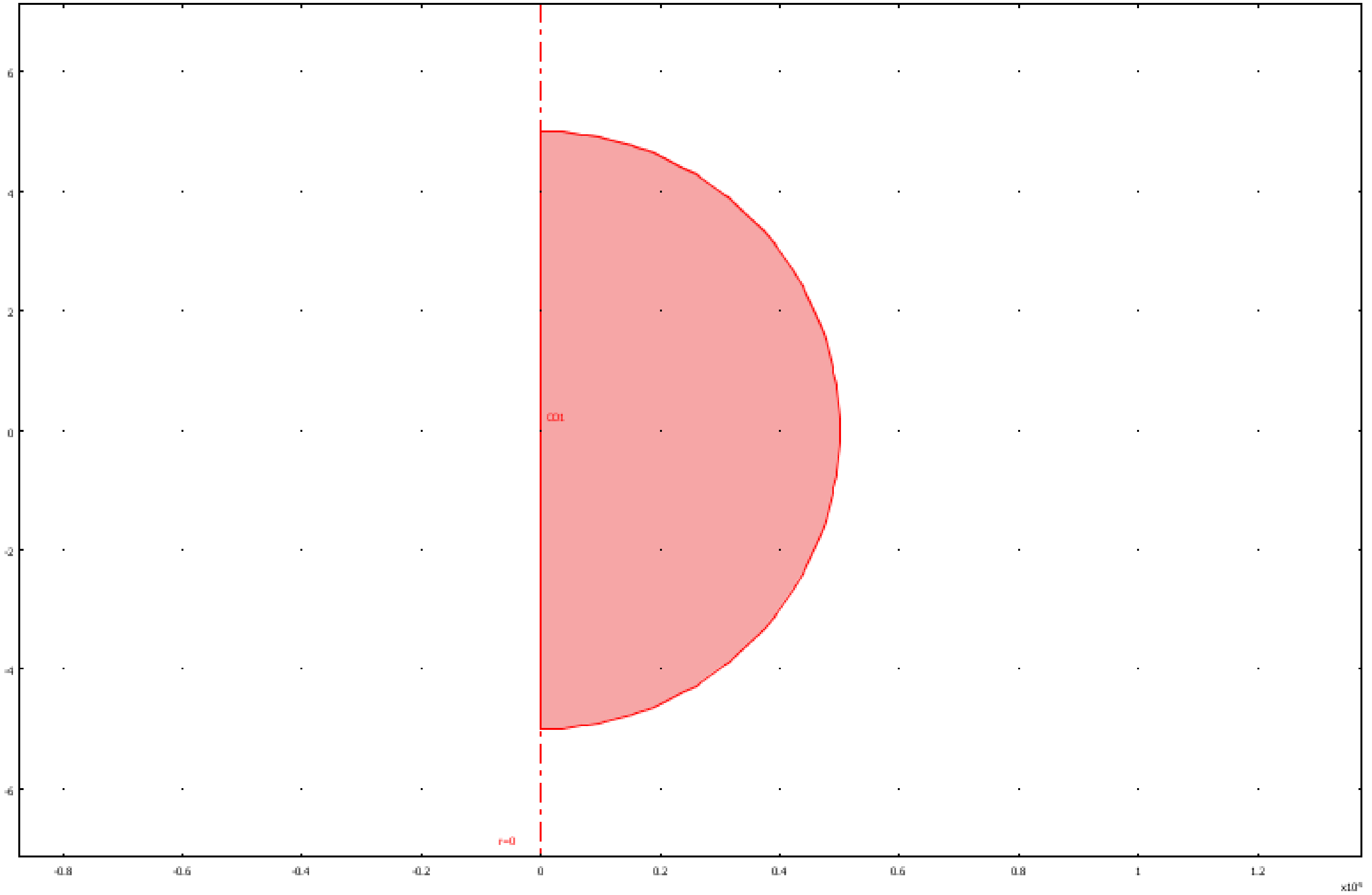

5.1.1. Point mode

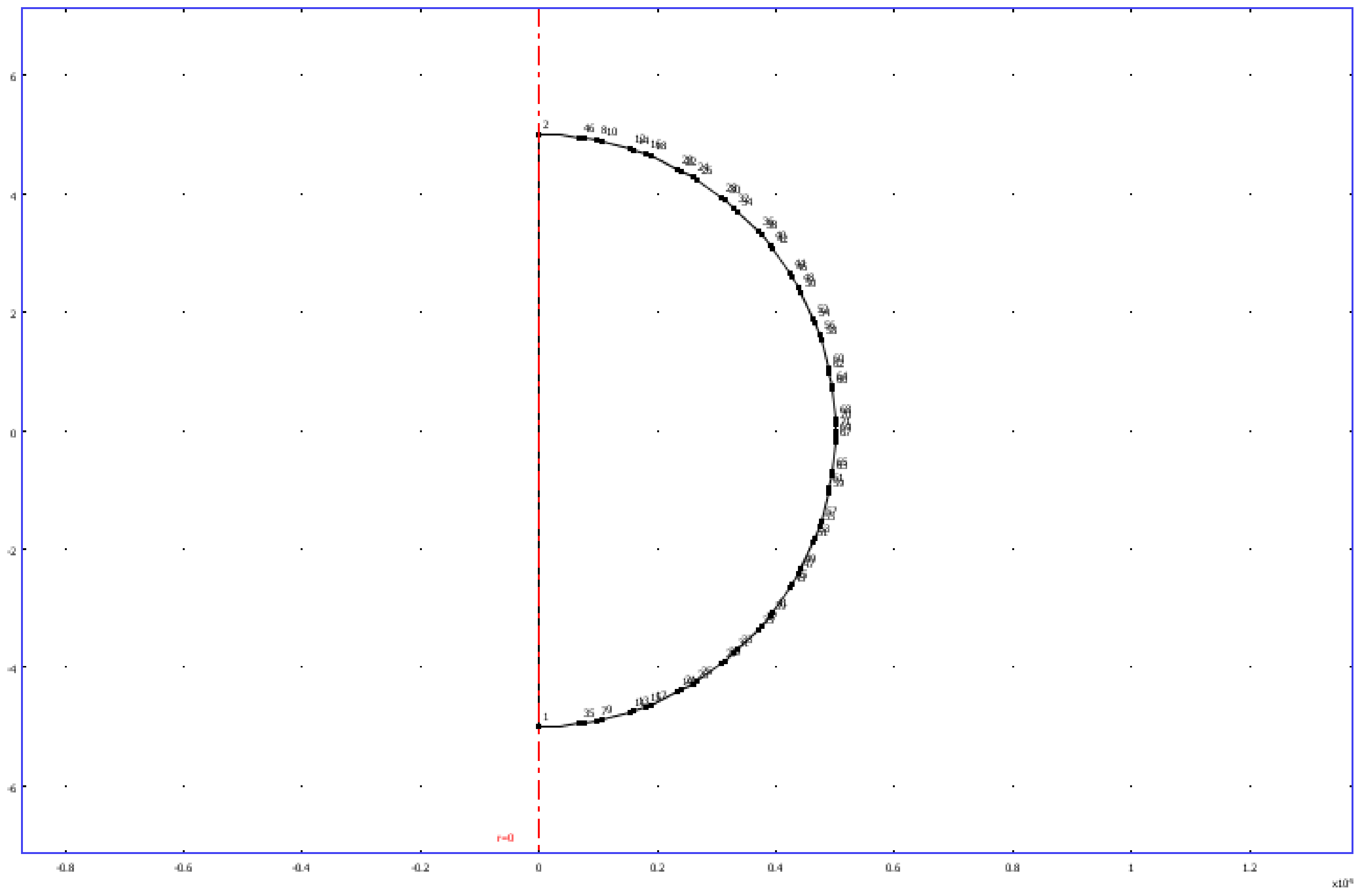

5.1.2. Boundary mode

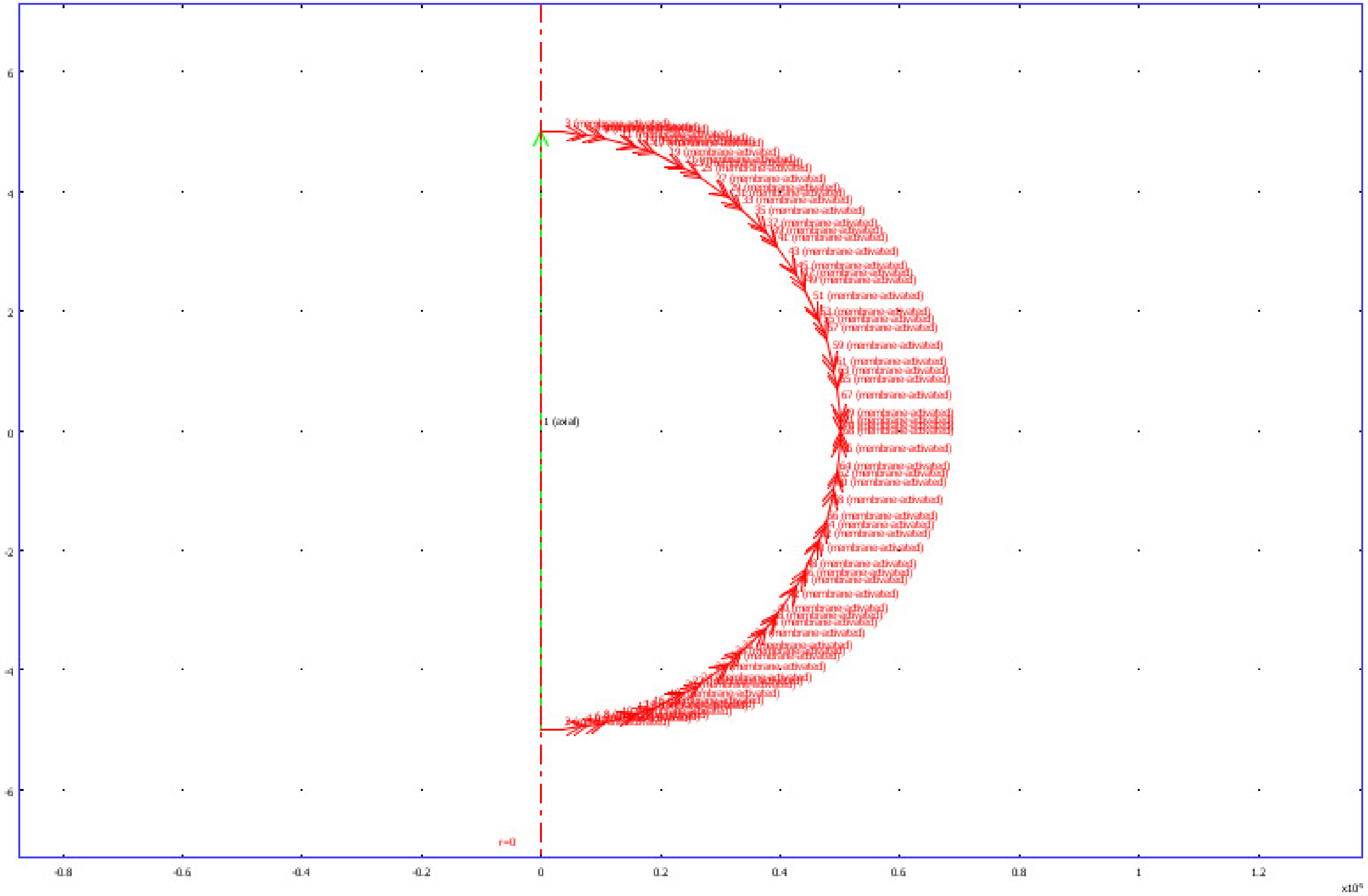

5.1.3. Subdomain mode

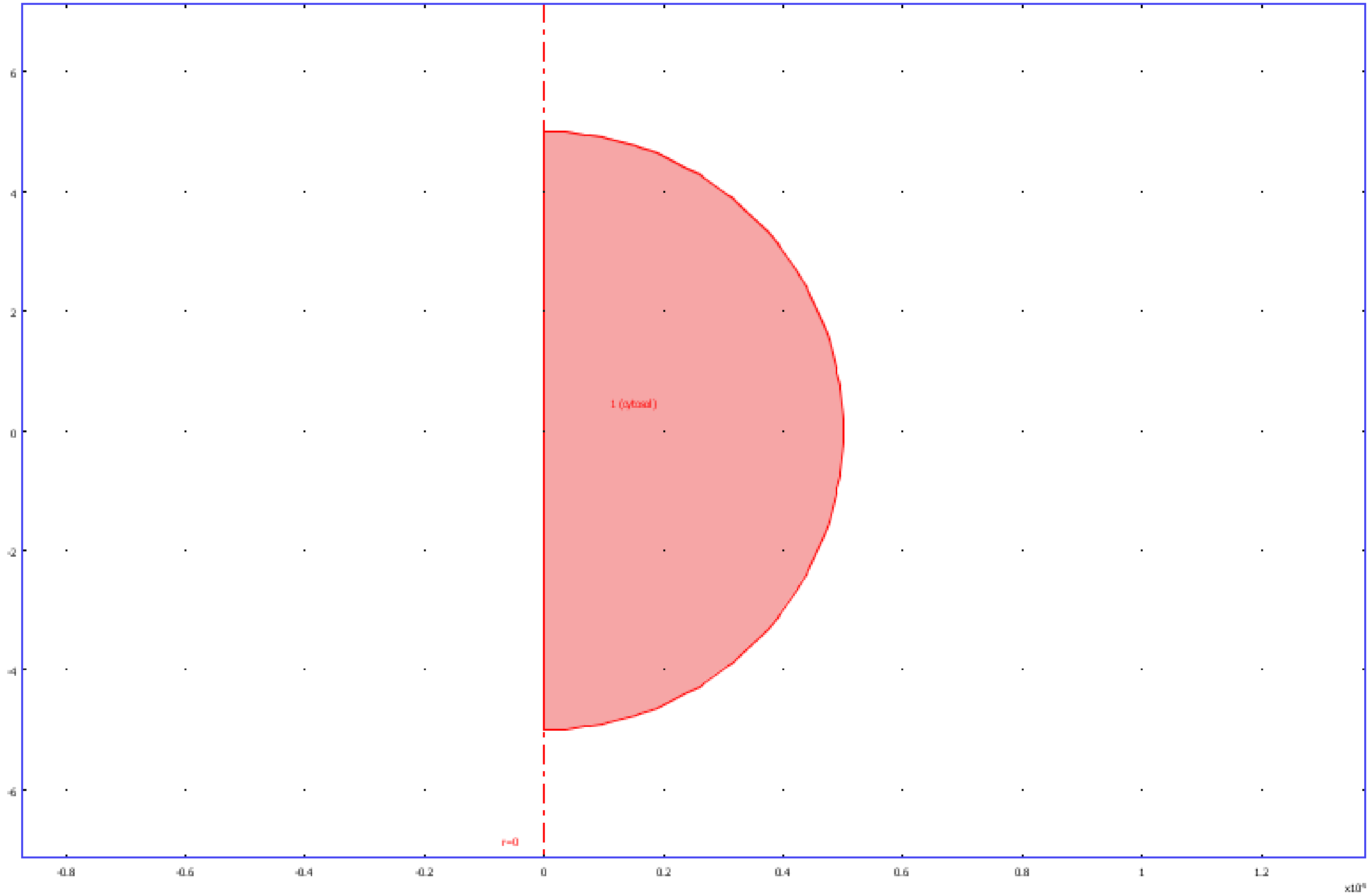

6. Geom1

Space dimensions: Axial symmetry (2D)

Independent variables: r, phi, z

6.1. Mesh

6.1.1. Mesh Statistics

Number of degrees of freedom	6546
Number of mesh points	583
Number of elements	1017
Triangular	1017
Quadrilateral	0
Number of boundary elements	147
Number of vertex elements	71
Minimum element quality	0.688
Element area ratio	0.001

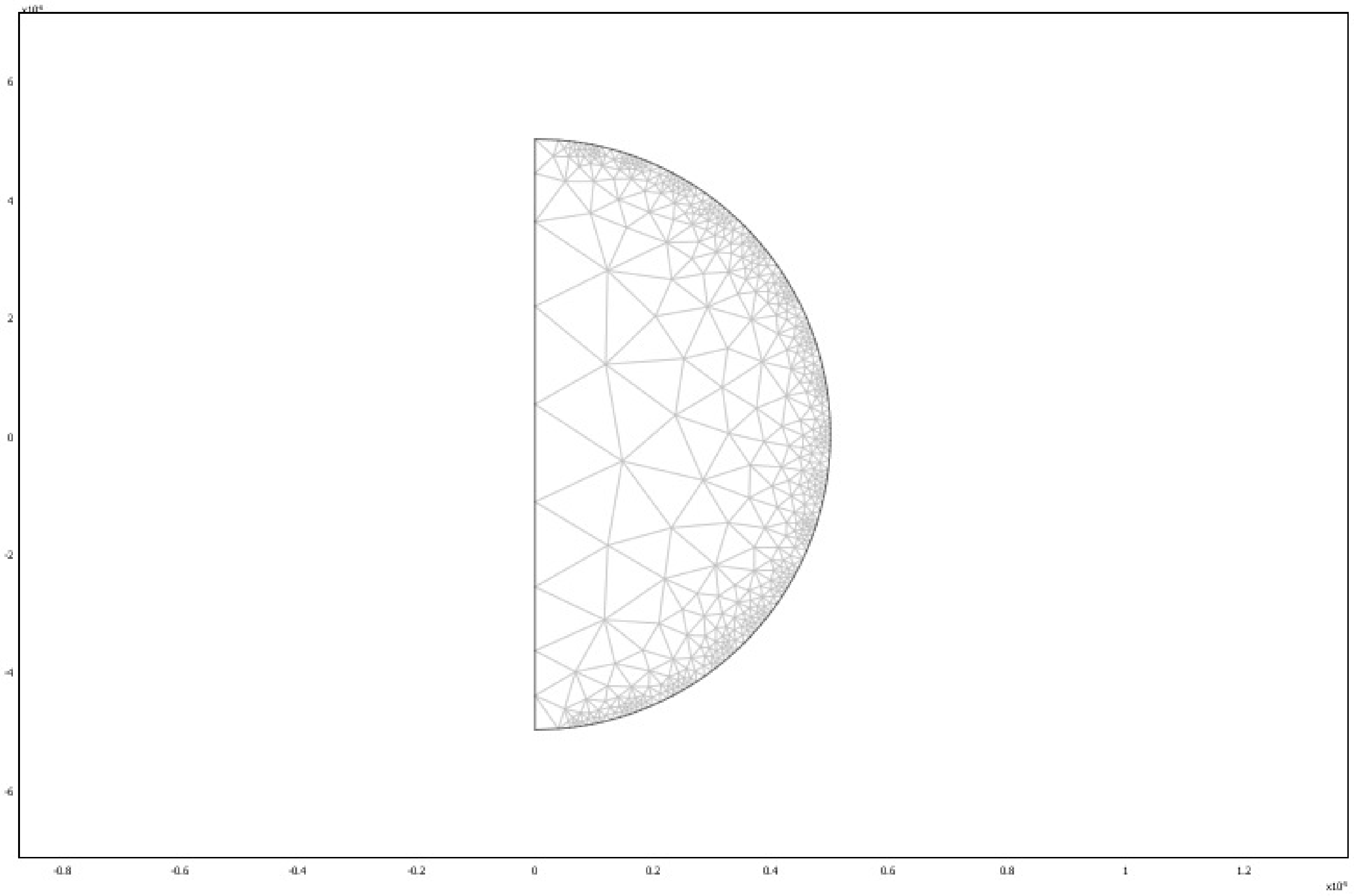

6.2. Application Mode: Diffusion (chdi)

Application mode type: Diffusion (Chemical Engineering Module)

Application mode name: chdi

6.2.1. Application Mode Properties

Property	Value
Default element type	Lagrange - Quadratic
Analysis type	Transient
Equilibrium assumption	Off
Frame	Frame (ref)
Weak constraints	Off
Constraint type	Ideal

6.2.2. Variables

Dependent variables: c_caFree, c_buffer, c_caBound

Shape functions: shlag(2,'c_caFree'), shlag(2,'c_buffer'), shlag(2,'c_caBound')

Interior boundaries not active

6.2.3. Boundary Settings

Boundary		1	2-71
Type		Axial symmetry	Flux
name		**axial**	**membrane-activated**
Inward flux (N)	$mol/(m^2 \cdot s)$	{0;0;0}	**{k_open*(Pstim/area)-Jefflux*c_caFree/(Km+c_caFree);0;0}**
Mass transfer coefficient (kc)	m/s	{0;0;0}	**{Prest;0;0}**
Bulk concentration (cb)	mol/m^3	{0;0;0}	**{CaExt;0;0}**

6.2.4. Subdomain Settings

Locked Subdomains: 1

Subdomain		1

name		**cytosol**
Diffusion coefficient (D)	m^2/s	**{D_Ca;D_buffer;D_Cabound}**
Reaction rate (R)	$mol/(m^3 \cdot s)$	**{-r_1_rxn_chdi;-r_1_rxn_chdi;r_1_rxn_chdi}**
(relExpr)		**{r_1_rxn;50000[m^3/(s*mol)]*c_caFree*c_buffer-25[1/s]*c_caBound}**

Subdomain initial value		1
Concentration, c_caFree (c_caFree)	mol/m^3	0.0001
Concentration, c_buffer (c_buffer)	mol/m^3	0.63333
Concentration, c_caBound (c_caBound)	mol/m^3	0.12667

7. Extrusion Coupling Variables

7.1. Geom1

7.1.1. Source Subdomain: 1

Name	Value
Expression	c_caFree*1e-3
Transformation type	General
Source transformation	r, z,
Name	Ca_3D

8. Solver Settings

Solve using a script: off

Analysis type	Transient
Auto select solver	On
Solver	Time dependent
Solution form	Automatic
Symmetric	auto

Adaptive mesh refinement	Off
Optimization/Sensitivity	Off
Plot while solving	Off

8.1. Direct (PARDISO)

Solver type: Linear system solver

Parameter	Value
Preordering algorithm	Nested dissection
Row preordering	On
Bunch-Kaufmann	Off
Pivoting perturbation	1.0E-8
Relative tolerance	1.0E-6
Factor in error estimate	400.0
Check tolerances	On

8.2. Time Stepping

Parameter	Value
Times	0:0.01:3
Relative tolerance	1e-7
Absolute tolerance	1e-8
Times to store in output	Specified times
Time steps taken by solver	Free
Maximum time step	.1
Maximum BDF order	5
Singular mass matrix	Maybe
Consistent initialization of DAE systems	Backward Euler
Error estimation strategy	Include algebraic
Allow complex numbers	Off

8.3. Advanced

Parameter	Value
Constraint handling method	Elimination
Null-space function	Automatic
Automatic assembly block size	On
Assembly block size	1000
Use Hermitian transpose of constraint matrix and in symmetry detection	Off
Use complex functions with real input	Off
Stop if error due to undefined operation	On
Store solution on file	Off
Type of scaling	Automatic
Manual scaling	
Row equilibration	On
Manual control of reassembly	Off
Load constant	On
Constraint constant	On
Mass constant	On
Damping (mass) constant	On
Jacobian constant	On
Constraint Jacobian constant	On

9. Postprocessing

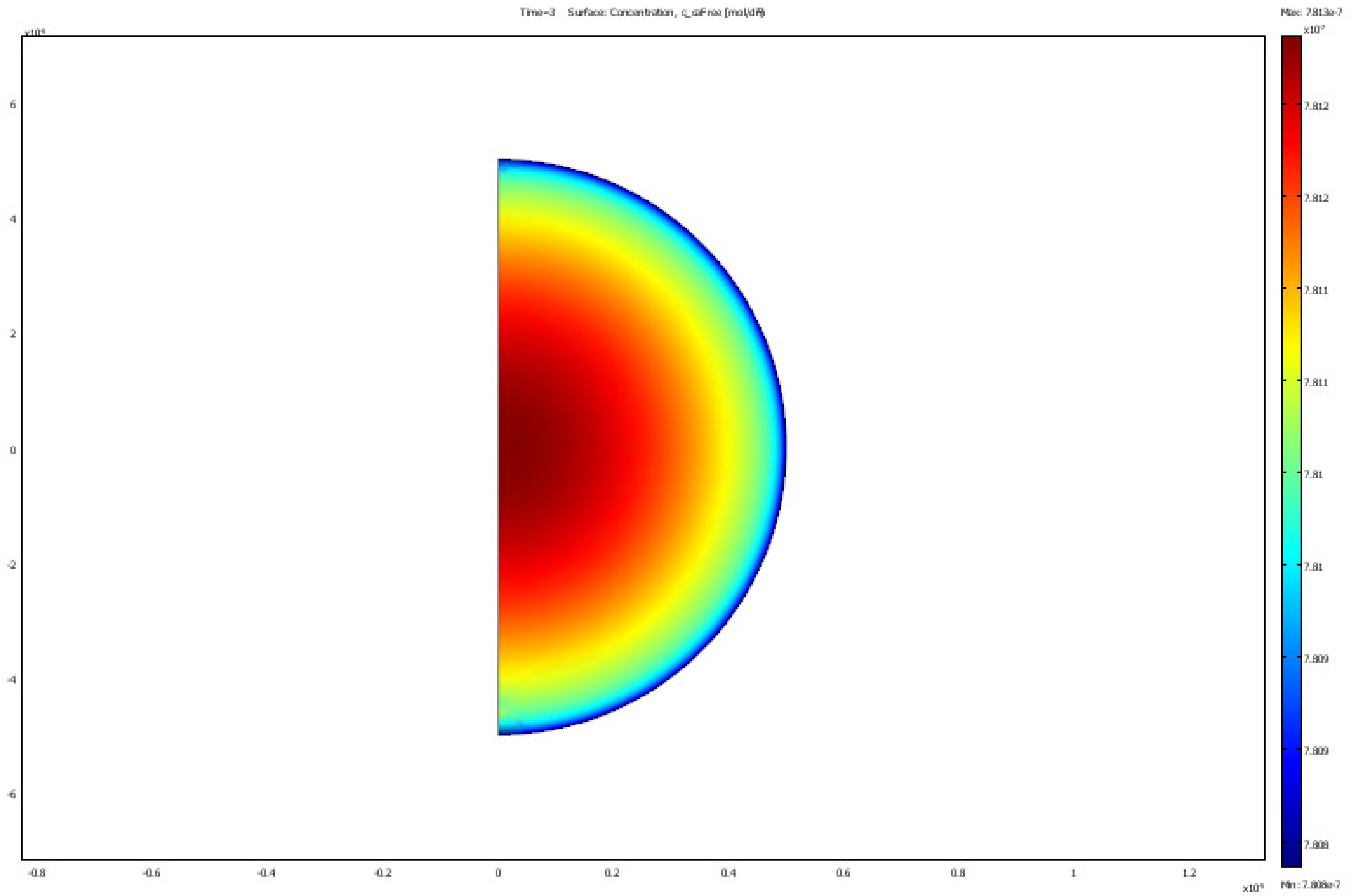

10. Equations

10.1. Subdomain

Dependent variables: c_caFree, c_buffer, c_caBound

10.1.1. Subdomain: 1

Diffusion coefficient (c)

c_caFree	c_buffer	c_caBound
-diff(r*(-Drr_c_caFree_chdi*c_caFreer-Drz_c_caFree_chdi*c_caFreez),c_caFreer), -diff(r*(-Dzr_c_caFree_chdi*c_caFreer-Dzz_c_caFree_chdi*c_caFreez),c_caFreer), -diff(r*(-Drr_c_caFree_chdi*c_caFreer-Drz_c_caFree_chdi*c_caFreez),c_caFreez), -diff(r*(-Dzr_c_caFree_chdi*c_caFreer-Dzz_c_caFree_chdi*c_caFreez),c_caFreez)	-diff(r*(-Drr_c_caFree_chdi*c_caFreer-Drz_c_caFree_chdi*c_caFreez),c_bufferr), -diff(r*(-Dzr_c_caFree_chdi*c_caFreer-Dzz_c_caFree_chdi*c_caFreez),c_bufferr), -diff(r*(-Drr_c_caFree_chdi*c_caFreer-Drz_c_caFree_chdi*c_caFreez),c_bufferz), -diff(r*(-Dzr_c_caFree_chdi*c_caFreer-Dzz_c_caFree_chdi*c_caFreez),c_bufferz)	-diff(r*(-Drr_c_caFree_chdi*c_caFreer-Drz_c_caFree_chdi*c_caFreez),c_caBoundr), -diff(r*(-Dzr_c_caFree_chdi*c_caFreer-Dzz_c_caFree_chdi*c_caFreez),c_caBoundr), -diff(r*(-Drr_c_caFree_chdi*c_caFreer-Drz_c_caFree_chdi*c_caFreez),c_caBoundz), -diff(r*(-Dzr_c_caFree_chdi*c_caFreer-Dzz_c_caFree_chdi*c_caFreez),c_caBoundz)
-diff(r*(-Drr_c_buffer_chdi*c_bufferr-Drz_c_buffer_chdi*c_bufferz),c_caFreer), -diff(r*(-Dzr_c_buffer_chdi*c_bufferr-Dzz_c_buffer_chdi*c_bufferz),c_caFreer), -diff(r*(-Drr_c_buffer_chdi*c_bufferr-Drz_c_buffer_chdi*c_bufferz),c_caFreez), -diff(r*(-Dzr_c_buffer_chdi*c_bufferr-Dzz_c_buffer_chdi*c_bufferz),c_caFreez)	-diff(r*(-Drr_c_buffer_chdi*c_bufferr-Drz_c_buffer_chdi*c_bufferz),c_bufferr), -diff(r*(-Dzr_c_buffer_chdi*c_bufferr-Dzz_c_buffer_chdi*c_bufferz),c_bufferr), -diff(r*(-Drr_c_buffer_chdi*c_bufferr-Drz_c_buffer_chdi*c_bufferz),c_bufferz), -diff(r*(-Dzr_c_buffer_chdi*c_bufferr-Dzz_c_buffer_chdi*c_bufferz),c_bufferz)	-diff(r*(-Drr_c_buffer_chdi*c_bufferr-Drz_c_buffer_chdi*c_bufferz),c_caBoundr), -diff(r*(-Dzr_c_buffer_chdi*c_bufferr-Dzz_c_buffer_chdi*c_bufferz),c_caBoundr), -diff(r*(-Drr_c_buffer_chdi*c_bufferr-Drz_c_buffer_chdi*c_bufferz),c_caBoundz), -diff(r*(-Dzr_c_buffer_chdi*c_bufferr-Dzz_c_buffer_chdi*c_bufferz),c_caBoundz)
-diff(r*(-Drr_c_caBound_chdi*c_caBoundr-Drz_c_caBound_chdi*c_caBoundz),c_caFreer), -diff(r*(-Dzr_c_caBound_chdi*c_caBoundr-Dzz_c_caBound_chdi*c_caBoundz),c_caFreer), -diff(r*(-Drr_c_caBound_chdi*c_caBoundr-Drz_c_caBound_chdi*c_caBoundz),c_caFreez), -diff(r*(-Dzr_c_caBound_chdi*c_caBoundr-Dzz_c_caBound_chdi*c_caBoundz),c_caFreez)	-diff(r*(-Drr_c_caBound_chdi*c_caBoundr-Drz_c_caBound_chdi*c_caBoundz),c_bufferr), -diff(r*(-Dzr_c_caBound_chdi*c_caBoundr-Dzz_c_caBound_chdi*c_caBoundz),c_bufferr), -diff(r*(-Drr_c_caBound_chdi*c_caBoundr-Drz_c_caBound_chdi*c_caBoundz),c_bufferz), -diff(r*(-Dzr_c_caBound_chdi*c_caBoundr-Dzz_c_caBound_chdi*c_caBoundz),c_bufferz)	-diff(r*(-Drr_c_caBound_chdi*c_caBoundr-Drz_c_caBound_chdi*c_caBoundz),c_caBoundr), -diff(r*(-Dzr_c_caBound_chdi*c_caBoundr-Dzz_c_caBound_chdi*c_caBoundz),c_caBoundr), -diff(r*(-Drr_c_caBound_chdi*c_caBoundr-Drz_c_caBound_chdi*c_caBoundz),c_caBoundz), -diff(r*(-Dzr_c_caBound_chdi*c_caBoundr-Dzz_c_caBound_chdi*c_caBoundz),c_caBoundz)

Absorption coefficient (a)

c_caFree	c_buffer	c_caBound
-diff(r*R_c_caFree_chdi,c_caFree)	-diff(r*R_c_caFree_chdi,c_buffer)	-diff(r*R_c_caFree_chdi,c_caBound)
-diff(r*R_c_buffer_chdi,c_caFree)	-diff(r*R_c_buffer_chdi,c_buffer)	-diff(r*R_c_buffer_chdi,c_caBound)

-diff(r*R_c_caBound_chdi,c_caFree)	-diff(r*R_c_caBound_chdi,c_buffer)	-diff(r*R_c_caBound_chdi,c_caBound)

Source term (f)

r*R_c_caFree_chdi
r*R_c_buffer_chdi
r*R_c_caBound_chdi

Damping/Mass coefficient (da)

c_caFree	c_buffer	c_caBound
r*Dts_c_caFree_chdi	0	0
0	r*Dts_c_buffer_chdi	0
0	0	r*Dts_c_caBound_chdi

Conservative flux convection coeff. (al)

c_caFree	c_buffer	c_caBound
-diff(r*(-Drr_c_caFree_chdi*c_caFreer-Drz_c_caFree_chdi*c_caFreez),c_caFree), -diff (r*(-Dzr_c_caFree_chdi*c_caFreer-Dzz_c_caFree_chdi*c_caFreez),c_caFree)	-diff(r*(-Drr_c_caFree_chdi*c_caFreer-Drz_c_caFree_chdi*c_caFreez),c_buffer), -diff (r*(-Dzr_c_caFree_chdi*c_caFreer-Dzz_c_caFree_chdi*c_caFreez),c_buffer)	-diff(r*(-Drr_c_caFree_chdi*c_caFreer-Drz_c_caFree_chdi*c_caFreez),c_caBound), -diff (r*(-Dzr_c_caFree_chdi*c_caFreer-Dzz_c_caFree_chdi*c_caFreez),c_caBound)
-diff(r*(-Drr_c_buffer_chdi*c_bufferr-Drz_c_buffer_chdi*c_bufferz),c_caFree), -diff(r* (-Dzr_c_buffer_chdi*c_bufferr-Dzz_c_buffer_chdi*c_bufferz),c_caFree)	-diff(r*(-Drr_c_buffer_chdi*c_bufferr-Drz_c_buffer_chdi*c_bufferz),c_buffer), -diff(r* (-Dzr_c_buffer_chdi*c_bufferr-Dzz_c_buffer_chdi*c_bufferz),c_buffer)	-diff(r*(-Drr_c_buffer_chdi*c_bufferr-Drz_c_buffer_chdi*c_bufferz),c_caBound), -diff(r* (-Dzr_c_buffer_chdi*c_bufferr-Dzz_c_buffer_chdi*c_bufferz),c_caBound)
-diff(r*(-Drr_c_caBound_chdi*c_caBoundr-Drz_c_caBound_chdi*c_caBoundz),c_caFree), -diff(r*(-Dzr_c_caBound_chdi*c_caBoundr-Dzz_c_caBound_chdi*c_caBoundz),c_caFree)	-diff(r*(-Drr_c_caBound_chdi*c_caBoundr-Drz_c_caBound_chdi*c_caBoundz),c_buffer), -diff(r*(-Dzr_c_caBound_chdi*c_caBoundr-Dzz_c_caBound_chdi*c_caBoundz),c_buffer)	-diff(r*(-Drr_c_caBound_chdi*c_caBoundr-Drz_c_caBound_chdi*c_caBoundz),c_caBound), -diff(r*(-Dzr_c_caBound_chdi*c_caBoundr-Dzz_c_caBound_chdi*c_caBoundz),c_caBound)

Convection coefficient (be)

c_caFree	c_buffer	c_caBound

-diff(r*R_c_caFree_chdi,c_caFreer), -diff (r*R_c_caFree_chdi,c_caFreez)	-diff(r*R_c_caFree_chdi,c_bufferr), -diff (r*R_c_caFree_chdi,c_bufferz)	-diff(r*R_c_caFree_chdi,c_caBoundr), -diff (r*R_c_caFree_chdi,c_caBoundz)
-diff(r*R_c_buffer_chdi,c_caFreer), -diff (r*R_c_buffer_chdi,c_caFreez)	-diff(r*R_c_buffer_chdi,c_bufferr), -diff (r*R_c_buffer_chdi,c_bufferz)	-diff(r*R_c_buffer_chdi,c_caBoundr), -diff (r*R_c_buffer_chdi,c_caBoundz)
-diff(r*R_c_caBound_chdi,c_caFreer), -diff (r*R_c_caBound_chdi,c_caFreez)	-diff(r*R_c_caBound_chdi,c_bufferr), -diff (r*R_c_caBound_chdi,c_bufferz)	-diff(r*R_c_caBound_chdi,c_caBoundr), -diff (r*R_c_caBound_chdi,c_caBoundz)

Conservative flux source term (ga)

r*(-Drr_c_caFree_chdi*c_caFreer-Drz_c_caFree_chdi*c_caFreez), r*(-Dzr_c_caFree_chdi*c_caFreer-Dzz_c_caFree_chdi*c_caFreez)
r*(-Drr_c_buffer_chdi*c_bufferr-Drz_c_buffer_chdi*c_bufferz), r*(-Dzr_c_buffer_chdi*c_bufferr-Dzz_c_buffer_chdi*c_bufferz)
r*(-Drr_c_caBound_chdi*c_caBoundr-Drz_c_caBound_chdi*c_caBoundz), r*(-Dzr_c_caBound_chdi*c_caBoundr-Dzz_c_caBound_chdi*c_caBoundz)

Initial value (init)

c_caFree	0.0001
c_buffer	0.63333
c_caBound	0.12667

11. Variables

11.1. Boundary

Name	Description	Unit	Expression
ndflux_c_caFree_chdi	Normal diffusive flux, c_caFree	mol/(m^2*s)	nr_chdi * dflux_c_caFree_r_chdi+nz_chdi * dflux_c_caFree_z_chdi
ndflux_c_buffer_chdi	Normal diffusive flux, c_buffer	mol/(m^2*s)	nr_chdi * dflux_c_buffer_r_chdi+nz_chdi * dflux_c_buffer_z_chdi
ndflux_c_caBound_chdi	Normal diffusive flux, c_caBound	mol/(m^2*s)	nr_chdi * dflux_c_caBound_r_chdi+nz_chdi * dflux_c_caBound_z_chdi

11.2. Subdomain

Name	Description	Unit	Expression

r_1_rxn_chdi	Reaction help variable	mol/(m^3*s)	kf * c_caFree * c_buffer-kr * c_caBound
grad_c_caFree_r_chdi	Concentration gradient, c_caFree, r component	mol/m^4	c_caFreer
dflux_c_caFree_r_chdi	Diffusive flux, c_caFree, r component	mol/(m^2*s)	-Drr_c_caFree_chdi * c_caFreer-Drz_c_caFree_chdi * c_caFreez
grad_c_caFree_z_chdi	Concentration gradient, c_caFree, z component	mol/m^4	c_caFreez
dflux_c_caFree_z_chdi	Diffusive flux, c_caFree, z component	mol/(m^2*s)	-Dzr_c_caFree_chdi * c_caFreer-Dzz_c_caFree_chdi * c_caFreez
grad_c_caFree_chdi	Concentration gradient, c_caFree	mol/m^4	sqrt(grad_c_caFree_r_chdi^2+grad_c_caFree_z_chdi^2)
dflux_c_caFree_chdi	Diffusive flux, c_caFree	mol/(m^2*s)	sqrt(dflux_c_caFree_r_chdi^2+dflux_c_caFree_z_chdi^2)
grad_c_buffer_r_chdi	Concentration gradient, c_buffer, r component	mol/m^4	c_bufferr
dflux_c_buffer_r_chdi	Diffusive flux, c_buffer, r component	mol/(m^2*s)	-Drr_c_buffer_chdi * c_bufferr-Drz_c_buffer_chdi * c_bufferz
grad_c_buffer_z_chdi	Concentration gradient, c_buffer, z component	mol/m^4	c_bufferz
dflux_c_buffer_z_chdi	Diffusive flux, c_buffer, z component	mol/(m^2*s)	-Dzr_c_buffer_chdi * c_bufferr-Dzz_c_buffer_chdi * c_bufferz
grad_c_buffer_chdi	Concentration gradient, c_buffer	mol/m^4	sqrt(grad_c_buffer_r_chdi^2+grad_c_buffer_z_chdi^2)
dflux_c_buffer_chdi	Diffusive flux, c_buffer	mol/(m^2*s)	sqrt(dflux_c_buffer_r_chdi^2+dflux_c_buffer_z_chdi^2)
grad_c_caBound_r_chdi	Concentration gradient, c_caBound, r component	mol/m^4	c_caBoundr
dflux_c_caBound_r_chdi	Diffusive flux, c_caBound, r component	mol/(m^2*s)	-Drr_c_caBound_chdi * c_caBoundr-Drz_c_caBound_chdi * c_caBoundz
grad_c_caBound_z_chdi	Concentration gradient, c_caBound, z component	mol/m^4	c_caBoundz
dflux_c_caBound_z_chdi	Diffusive flux, c_caBound, z component	mol/(m^2*s)	-Dzr_c_caBound_chdi * c_caBoundr-Dzz_c_caBound_chdi * c_caBoundz
grad_c_caBound_chdi	Concentration gradient, c_caBound	mol/m^4	sqrt(grad_c_caBound_r_chdi^2+grad_c_caBound_z_chdi^2)
dflux_c_caBound_chdi	Diffusive flux, c_caBound	mol/(m^2*s)	sqrt(dflux_c_caBound_r_chdi^2+dflux_c_caBound_z_chdi^2)

COMSOL Model Report

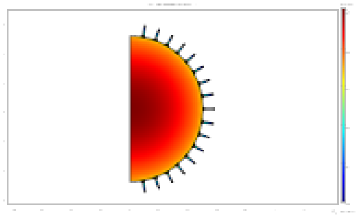

1. Table of Contents

- Title - COMSOL Model Report
- Table of Contents
- Model Properties
- Postprocessing
- Geom1
- Global Expressions
- Constants
- Extrusion Coupling Variables
- Solver Settings
- Equations
- Variables

2. Model Properties

Property	Value
Model name	

Author	
Company	
Department	
Reference	
URL	
Saved date	Mar 12, 2009 2:52:19 PM
Creation date	Dec 2, 2008 10:12:36 AM
COMSOL version	COMSOL 3.5.0.494

File name: C:\20090312-wrinkles.mph

Application modes and modules used in this model:

- Geom1 (Axial symmetry (2D))
  - Diffusion (Chemical Engineering Module)

3. Postprocessing

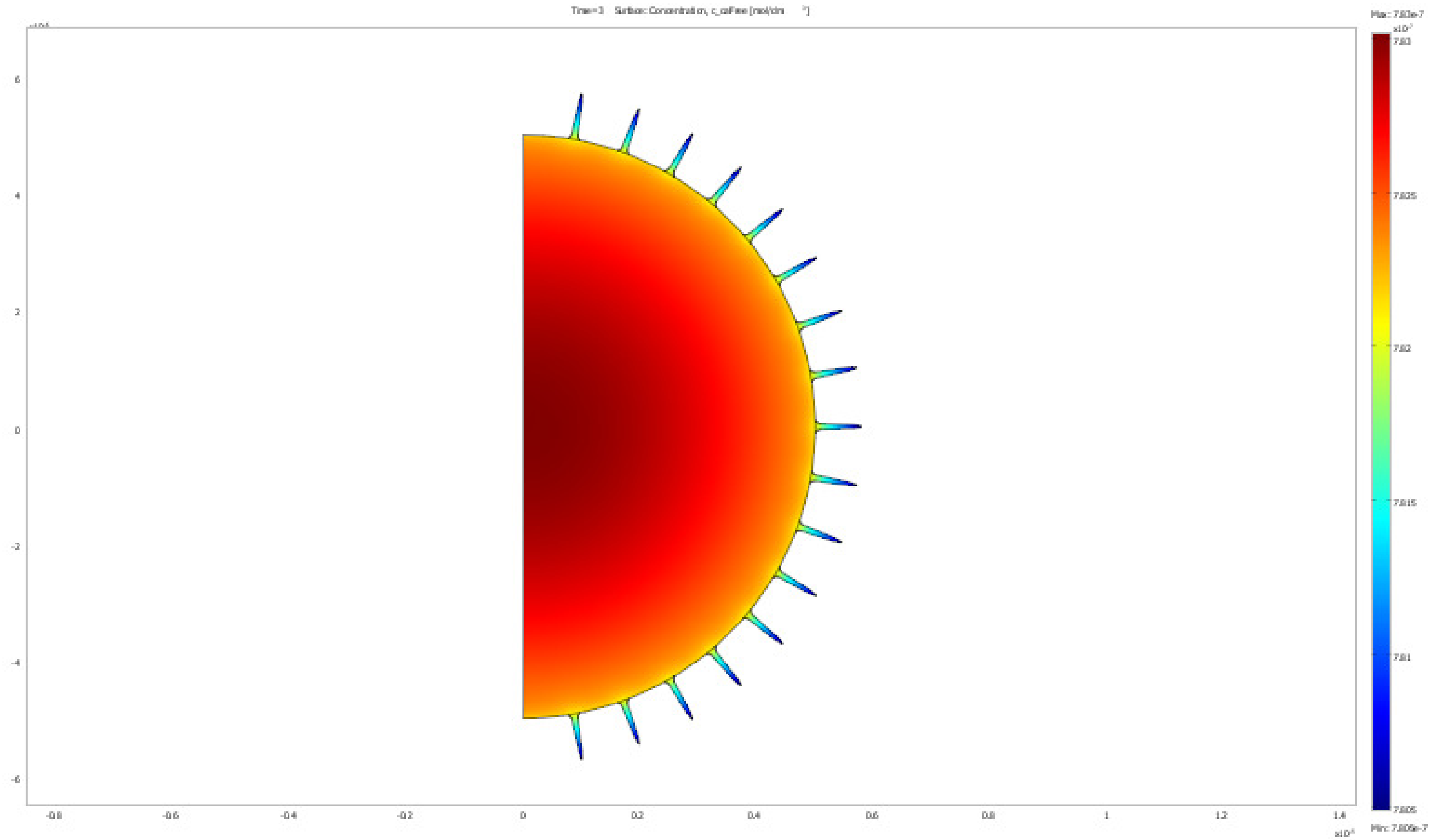

4. Geom1

Space dimensions: Axial symmetry (2D)

Independent variables: r, phi, z

4.1. Mesh

4.1.1. Mesh Statistics

Number of degrees of freedom	37707
Number of mesh points	3372
Number of elements	5826
Triangular	5826
Quadrilateral	0
Number of boundary elements	1032
Number of vertex elements	122
Minimum element quality	0.725
Element area ratio	0

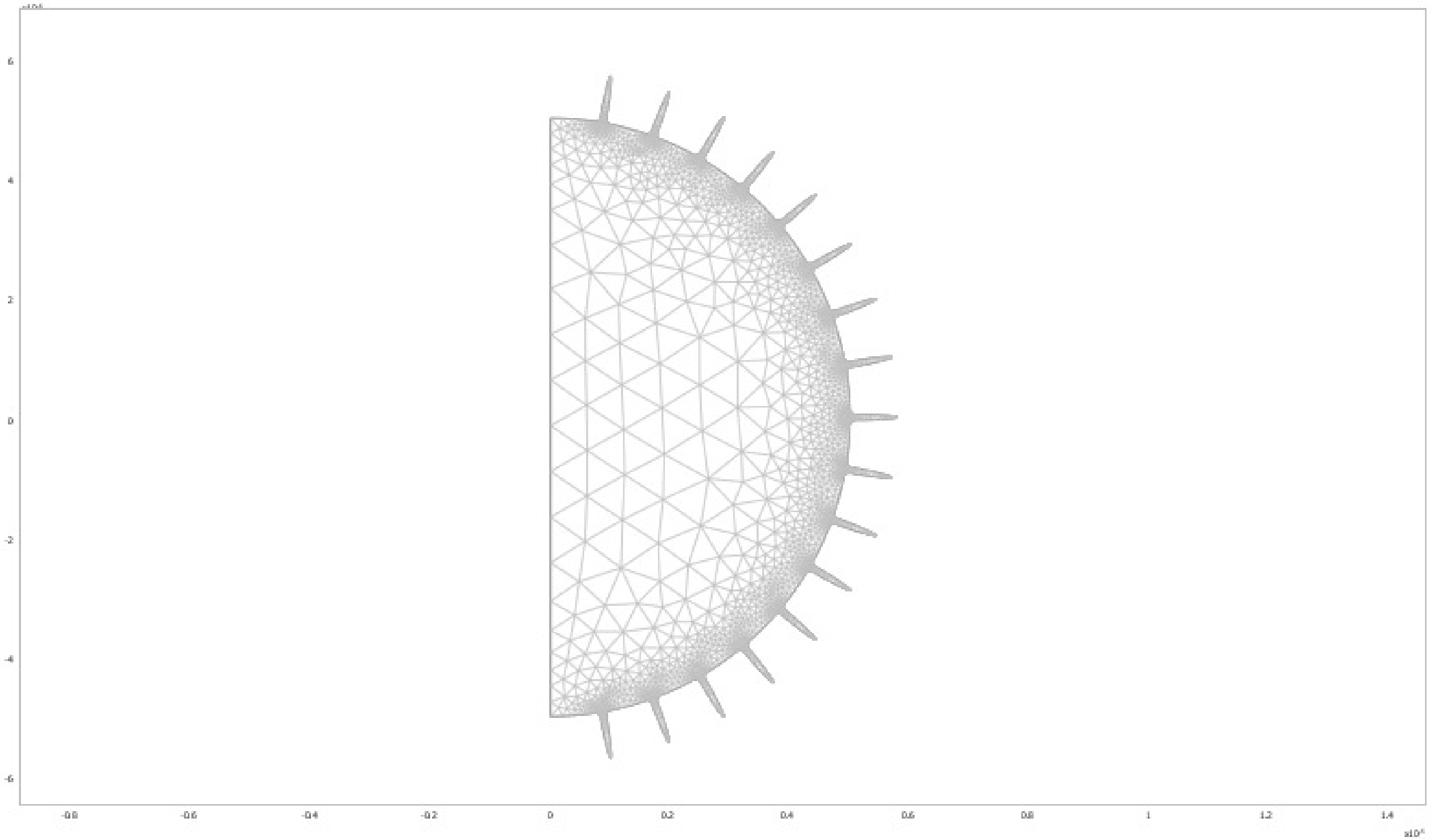

4.2. Application Mode: Diffusion (chdi)

Application mode type: Diffusion (Chemical Engineering Module)

Application mode name: chdi

4.2.1. Application Mode Properties

Property	Value
Default element type	Lagrange - Quadratic
Analysis type	Transient
Equilibrium assumption	Off
Frame	Frame (ref)
Weak constraints	Off
Constraint type	Ideal

4.2.2. Variables

Dependent variables: c_caFree, c_buffer, c_caBound

Shape functions: shlag(2,'c_caFree'), shlag(2,'c_buffer'), shlag(2,'c_caBound')

Interior boundaries not active

4.2.3. Boundary Settings

Boundary		2-6, 8, 10-22, 24, 26-38, 40, 42-54, 56, 58-70, 72, 74-86, 88, 90-101, 103, 105-117, 119, 121-129, 131, 133-139	1
Type		Flux	Axial symmetry
name		**membrane-activated**	**axial**
Inward flux (N)	mol/($m^2 \cdot s$)	**{k_open*(Pstim/area)-Jefflux*c_caFree/(Km+c_caFree);0;0}**	{0;0;0}
Mass transfer coefficient (kc)	m/s	**{Prest;0;0}**	{0;0;0}
Bulk concentration (cb)	mol/m^3	**{CaExt;0;0}**	{0;0;0}

4.2.4. Subdomain Settings

Locked Subdomains: 1-18

Subdomain		1-18
name		**cytosol**
Diffusion coefficient (D)	m^2/s	**{D_Ca;D_buffer;D_Cabound}**
Reaction rate (R)	$mol/(m^3 \cdot s)$	**{-r_1_rxn_chdi;-r_1_rxn_chdi;r_1_rxn_chdi}**
(relExpr)		**{r_1_rxn;50000[m^3/(s*mol)]*c_caFree*c_buffer-25[1/s]*c_caBound}**

Subdomain initial value		1-18
Concentration, c_caFree (c_caFree)	mol/m^3	0.0001
Concentration, c_buffer (c_buffer)	mol/m^3	0.63333
Concentration, c_caBound (c_caBound)	mol/m^3	0.12667

5. Global Expressions

Name	Expression	Unit	Description
k_open	200*(flc2hs(t-start,steep)-flc2hs(t-stop,steep))		

6. Constants

Name	Expression	Value	Description
D_Ca	233[um^2/s]	(2.33e-10)[m^2/s]	
D_buffer	13[um^2/s]	(1.3e-11)[m^2/s]	
D_Cabound	13[um^2/s]	(1.3e-11)[m^2/s]	
start	1[s]	1[s]	
stop	2[s]	2[s]	
steep	.001	0.001	
Pstim	9.3e-19[mol/(s)]	(9.3e-19)[mol/s]	
area	8.706643e-10 [m^2]	(8.706643e-10)[m^2]	
Prest	8e-9[cm/(s)]	(8e-11)[m/s]	

Jefflux	1.28e-9 [mol/m^2/s]	(1.28e-9)[mol/($m^2 \cdot s$)]	
CaExt	1[mmol/liter]	1[mol/m^3]	
Km	1.5[umol/liter]	0.0015[mol/m^3]	
kf	(50/1e-6)[1/((mol/liter)*s)]	50000[m^3/($s \cdot mol$)]	
kr	25[1/s]	25[1/s]	

7. Extrusion Coupling Variables

7.1. Geom1

7.1.1. Source Subdomain: 1-18

**Name**	**Value**
Expression	c_caFree*1e-3
Transformation type	General
Source transformation	r, z,
Name	ca3d

8. Solver Settings

Solve using a script: off

Analysis type	Transient
Auto select solver	On
Solver	Time dependent
Solution form	Automatic
Symmetric	auto
Adaptive mesh refinement	Off
Optimization/Sensitivity	Off
Plot while solving	Off

8.1. Direct (PARDISO)

Solver type: Linear system solver

Parameter	Value
Preordering algorithm	Nested dissection
Row preordering	On
Bunch-Kaufmann	Off
Pivoting perturbation	1.0E-8
Relative tolerance	1.0E-6
Factor in error estimate	400.0
Check tolerances	On

8.2. Time Stepping

Parameter	Value
Times	0:0.01:3
Relative tolerance	1e-7
Absolute tolerance	1e-8
Times to store in output	Specified times
Time steps taken by solver	Free
Maximum time step	.1
Maximum BDF order	5
Singular mass matrix	Maybe
Consistent initialization of DAE systems	Backward Euler
Error estimation strategy	Include algebraic
Allow complex numbers	Off

8.3. Advanced

Parameter	Value
Constraint handling method	Elimination

Null-space function	Automatic
Automatic assembly block size	On
Assembly block size	1000
Use Hermitian transpose of constraint matrix and in symmetry detection	Off
Use complex functions with real input	Off
Stop if error due to undefined operation	On
Store solution on file	Off
Type of scaling	Automatic
Manual scaling	
Row equilibration	On
Manual control of reassembly	Off
Load constant	On
Constraint constant	On
Mass constant	On
Damping (mass) constant	On
Jacobian constant	On
Constraint Jacobian constant	On

9. Equations

9.1. Subdomain

Dependent variables: c_caFree, c_buffer, c_caBound

9.1.1. Subdomain: 1-18

Diffusion coefficient (c)

c_caFree	c_buffer	c_caBound
-diff(r*(-Drr_c_caFree_chdi*c_caFreer-Drz_c_caFree_chdi*c_caFreez),c_caFreer), -diff (r*(-Dzr_c_caFree_chdi*c_caFreer-Dzz_c_caFree_chdi*c_caFreez),c_caFreer), -	-diff(r*(-Drr_c_caFree_chdi*c_caFreer-Drz_c_caFree_chdi*c_caFreez),c_bufferr), -diff (r*(-Dzr_c_caFree_chdi*c_caFreer-Dzz_c_caFree_chdi*c_caFreez),c_bufferr), -	-diff(r*(-Drr_c_caFree_chdi*c_caFreer-Drz_c_caFree_chdi*c_caFreez),c_caBoundr), -diff (r*(-Dzr_c_caFree_chdi*c_caFreer-Dzz_c_caFree_chdi*c_caFreez),c_caBoundr), -

diff(r*(-Drr_c_caFree_chdi*c_caFreer-Drz_c_caFree_chdi*c_caFreez),c_caFreez), -diff(r*(-Dzr_c_caFree_chdi*c_caFreer-Dzz_c_caFree_chdi*c_caFreez),c_caFreez)	diff(r*(-Drr_c_caFree_chdi*c_caFreer-Drz_c_caFree_chdi*c_caFreez),c_bufferz), -diff(r*(-Dzr_c_caFree_chdi*c_caFreer-Dzz_c_caFree_chdi*c_caFreez),c_bufferz)	diff(r*(-Drr_c_caFree_chdi*c_caFreer-Drz_c_caFree_chdi*c_caFreez),c_caBoundz), -diff(r*(-Dzr_c_caFree_chdi*c_caFreer-Dzz_c_caFree_chdi*c_caFreez),c_caBoundz)
-diff(r*(-Drr_c_buffer_chdi*c_bufferr-Drz_c_buffer_chdi*c_bufferz),c_caFreer), -diff(r*(-Dzr_c_buffer_chdi*c_bufferr-Dzz_c_buffer_chdi*c_bufferz),c_caFreer), -diff(r*(-Drr_c_buffer_chdi*c_bufferr-Drz_c_buffer_chdi*c_bufferz),c_caFreez), -diff(r*(-Dzr_c_buffer_chdi*c_bufferr-Dzz_c_buffer_chdi*c_bufferz),c_caFreez)	-diff(r*(-Drr_c_buffer_chdi*c_bufferr-Drz_c_buffer_chdi*c_bufferz),c_bufferr), -diff(r*(-Dzr_c_buffer_chdi*c_bufferr-Dzz_c_buffer_chdi*c_bufferz),c_bufferr), -diff(r*(-Drr_c_buffer_chdi*c_bufferr-Drz_c_buffer_chdi*c_bufferz),c_bufferz), -diff(r*(-Dzr_c_buffer_chdi*c_bufferr-Dzz_c_buffer_chdi*c_bufferz),c_bufferz)	-diff(r*(-Drr_c_buffer_chdi*c_bufferr-Drz_c_buffer_chdi*c_bufferz),c_caBoundr), -diff(r*(-Dzr_c_buffer_chdi*c_bufferr-Dzz_c_buffer_chdi*c_bufferz),c_caBoundr), -diff(r*(-Drr_c_buffer_chdi*c_bufferr-Drz_c_buffer_chdi*c_bufferz),c_caBoundz), -diff(r*(-Dzr_c_buffer_chdi*c_bufferr-Dzz_c_buffer_chdi*c_bufferz),c_caBoundz)
-diff(r*(-Drr_c_caBound_chdi*c_caBoundr-Drz_c_caBound_chdi*c_caBoundz),c_caFreer), -diff(r*(-Dzr_c_caBound_chdi*c_caBoundr-Dzz_c_caBound_chdi*c_caBoundz),c_caFreer), -diff(r*(-Drr_c_caBound_chdi*c_caBoundr-Drz_c_caBound_chdi*c_caBoundz),c_caFreez), -diff(r*(-Dzr_c_caBound_chdi*c_caBoundr-Dzz_c_caBound_chdi*c_caBoundz),c_caFreez)	-diff(r*(-Drr_c_caBound_chdi*c_caBoundr-Drz_c_caBound_chdi*c_caBoundz),c_bufferr), -diff(r*(-Dzr_c_caBound_chdi*c_caBoundr-Dzz_c_caBound_chdi*c_caBoundz),c_bufferr), -diff(r*(-Drr_c_caBound_chdi*c_caBoundr-Drz_c_caBound_chdi*c_caBoundz),c_bufferz), -diff(r*(-Dzr_c_caBound_chdi*c_caBoundr-Dzz_c_caBound_chdi*c_caBoundz),c_bufferz)	-diff(r*(-Drr_c_caBound_chdi*c_caBoundr-Drz_c_caBound_chdi*c_caBoundz),c_caBoundr), -diff(r*(-Dzr_c_caBound_chdi*c_caBoundr-Dzz_c_caBound_chdi*c_caBoundz),c_caBoundr), -diff(r*(-Drr_c_caBound_chdi*c_caBoundr-Drz_c_caBound_chdi*c_caBoundz),c_caBoundz), -diff(r*(-Dzr_c_caBound_chdi*c_caBoundr-Dzz_c_caBound_chdi*c_caBoundz),c_caBoundz)

Absorption coefficient (a)

c_caFree	c_buffer	c_caBound
-diff(r*R_c_caFree_chdi,c_caFree)	-diff(r*R_c_caFree_chdi,c_buffer)	-diff(r*R_c_caFree_chdi,c_caBound)
-diff(r*R_c_buffer_chdi,c_caFree)	-diff(r*R_c_buffer_chdi,c_buffer)	-diff(r*R_c_buffer_chdi,c_caBound)
-diff(r*R_c_caBound_chdi,c_caFree)	-diff(r*R_c_caBound_chdi,c_buffer)	-diff(r*R_c_caBound_chdi,c_caBound)

Source term (f)

r*R_c_caFree_chdi
r*R_c_buffer_chdi
r*R_c_caBound_chdi

Damping/Mass coefficient (da)

c_caFree	c_buffer	c_caBound
r*Dts_c_caFree_chdi	0	0
0	r*Dts_c_buffer_chdi	0
0	0	r*Dts_c_caBound_chdi

Conservative flux convection coeff. (al)

c_caFree	c_buffer	c_caBound
-diff(r*(-Drr_c_caFree_chdi*c_caFreer-Drz_c_caFree_chdi*c_caFreez),c_caFree), -diff (r*(-Dzr_c_caFree_chdi*c_caFreer-Dzz_c_caFree_chdi*c_caFreez),c_caFree)	-diff(r*(-Drr_c_caFree_chdi*c_caFreer-Drz_c_caFree_chdi*c_caFreez),c_buffer), -diff (r*(-Dzr_c_caFree_chdi*c_caFreer-Dzz_c_caFree_chdi*c_caFreez),c_buffer)	-diff(r*(-Drr_c_caFree_chdi*c_caFreer-Drz_c_caFree_chdi*c_caFreez),c_caBound), -diff (r*(-Dzr_c_caFree_chdi*c_caFreer-Dzz_c_caFree_chdi*c_caFreez),c_caBound)
-diff(r*(-Drr_c_buffer_chdi*c_bufferr-Drz_c_buffer_chdi*c_bufferz),c_caFree), -diff(r* (-Dzr_c_buffer_chdi*c_bufferr-Dzz_c_buffer_chdi*c_bufferz),c_caFree)	-diff(r*(-Drr_c_buffer_chdi*c_bufferr-Drz_c_buffer_chdi*c_bufferz),c_buffer), -diff(r* (-Dzr_c_buffer_chdi*c_bufferr-Dzz_c_buffer_chdi*c_bufferz),c_buffer)	-diff(r*(-Drr_c_buffer_chdi*c_bufferr-Drz_c_buffer_chdi*c_bufferz),c_caBound), -diff(r* (-Dzr_c_buffer_chdi*c_bufferr-Dzz_c_buffer_chdi*c_bufferz),c_caBound)
-diff(r*(-Drr_c_caBound_chdi*c_caBoundr-Drz_c_caBound_chdi*c_caBoundz),c_caFree), -diff(r*(-Dzr_c_caBound_chdi*c_caBoundr-Dzz_c_caBound_chdi*c_caBoundz),c_caFree)	-diff(r*(-Drr_c_caBound_chdi*c_caBoundr-Drz_c_caBound_chdi*c_caBoundz),c_buffer), -diff(r*(-Dzr_c_caBound_chdi*c_caBoundr-Dzz_c_caBound_chdi*c_caBoundz),c_buffer)	-diff(r*(-Drr_c_caBound_chdi*c_caBoundr-Drz_c_caBound_chdi*c_caBoundz),c_caBound), -diff(r*(-Dzr_c_caBound_chdi*c_caBoundr-Dzz_c_caBound_chdi*c_caBoundz),c_caBound)

Convection coefficient (be)

c_caFree	c_buffer	c_caBound
-diff(r*R_c_caFree_chdi,c_caFreer), -diff (r*R_c_caFree_chdi,c_caFreez)	-diff(r*R_c_caFree_chdi,c_bufferr), -diff (r*R_c_caFree_chdi,c_bufferz)	-diff(r*R_c_caFree_chdi,c_caBoundr), -diff (r*R_c_caFree_chdi,c_caBoundz)
-diff(r*R_c_buffer_chdi,c_caFreer), -diff (r*R_c_buffer_chdi,c_caFreez)	-diff(r*R_c_buffer_chdi,c_bufferr), -diff (r*R_c_buffer_chdi,c_bufferz)	-diff(r*R_c_buffer_chdi,c_caBoundr), -diff (r*R_c_buffer_chdi,c_caBoundz)
-diff(r*R_c_caBound_chdi,c_caFreer), -diff (r*R_c_caBound_chdi,c_caFreez)	-diff(r*R_c_caBound_chdi,c_bufferr), -diff (r*R_c_caBound_chdi,c_bufferz)	-diff(r*R_c_caBound_chdi,c_caBoundr), -diff (r*R_c_caBound_chdi,c_caBoundz)

Conservative flux source term (ga)

r*(-Drr_c_caFree_chdi*c_caFreer-Drz_c_caFree_chdi*c_caFreez), r*(-Dzr_c_caFree_chdi*c_caFreer-Dzz_c_caFree_chdi*c_caFreez)
r*(-Drr_c_buffer_chdi*c_bufferr-Drz_c_buffer_chdi*c_bufferz), r*(-Dzr_c_buffer_chdi*c_bufferr-Dzz_c_buffer_chdi*c_bufferz)

r*(-Drr_c_caBound_chdi*c_caBoundr-Drz_c_caBound_chdi*c_caBoundz), r*(-Dzr_c_caBound_chdi*c_caBoundr-Dzz_c_caBound_chdi*c_caBoundz)

Initial value (init)

c_caFree	0.0001
c_buffer	0.63333
c_caBound	0.12667

10. Variables

10.1. Boundary

Name	Description	Unit	Expression
ndflux_c_caFree_chdi	Normal diffusive flux, c_caFree	mol/(m^2*s)	nr_chdi * dflux_c_caFree_r_chdi+nz_chdi * dflux_c_caFree_z_chdi
ndflux_c_buffer_chdi	Normal diffusive flux, c_buffer	mol/(m^2*s)	nr_chdi * dflux_c_buffer_r_chdi+nz_chdi * dflux_c_buffer_z_chdi
ndflux_c_caBound_chdi	Normal diffusive flux, c_caBound	mol/(m^2*s)	nr_chdi * dflux_c_caBound_r_chdi+nz_chdi * dflux_c_caBound_z_chdi

10.2. Subdomain

Name	Description	Unit	Expression
r_1_rxn_chdi	Reaction help variable	mol/(m^3*s)	kf * c_caFree * c_buffer-kr * c_caBound
grad_c_caFree_r_chdi	Concentration gradient, c_caFree, r component	mol/m^4	c_caFreer
dflux_c_caFree_r_chdi	Diffusive flux, c_caFree, r component	mol/(m^2*s)	-Drr_c_caFree_chdi * c_caFreer-Drz_c_caFree_chdi * c_caFreez
grad_c_caFree_z_chdi	Concentration gradient, c_caFree, z component	mol/m^4	c_caFreez
dflux_c_caFree_z_chdi	Diffusive flux, c_caFree, z component	mol/(m^2*s)	-Dzr_c_caFree_chdi * c_caFreer-Dzz_c_caFree_chdi * c_caFreez
grad_c_caFree_chdi	Concentration gradient, c_caFree	mol/m^4	sqrt(grad_c_caFree_r_chdi^2+grad_c_caFree_z_chdi^2)
dflux_c_caFree_chdi	Diffusive flux, c_caFree	mol/	sqrt(dflux_c_caFree_r_chdi^2+dflux_c_caFree_z_chdi^2)

		(m^2*s)	
grad_c_buffer_r_chdi	Concentration gradient, c_buffer, r component	mol/m^4	c_bufferr
dflux_c_buffer_r_chdi	Diffusive flux, c_buffer, r component	mol/(m^2*s)	-Drr_c_buffer_chdi * c_bufferr-Drz_c_buffer_chdi * c_bufferz
grad_c_buffer_z_chdi	Concentration gradient, c_buffer, z component	mol/m^4	c_bufferz
dflux_c_buffer_z_chdi	Diffusive flux, c_buffer, z component	mol/(m^2*s)	-Dzr_c_buffer_chdi * c_bufferr-Dzz_c_buffer_chdi * c_bufferz
grad_c_buffer_chdi	Concentration gradient, c_buffer	mol/m^4	sqrt(grad_c_buffer_r_chdi^2+grad_c_buffer_z_chdi^2)
dflux_c_buffer_chdi	Diffusive flux, c_buffer	mol/(m^2*s)	sqrt(dflux_c_buffer_r_chdi^2+dflux_c_buffer_z_chdi^2)
grad_c_caBound_r_chdi	Concentration gradient, c_caBound, r component	mol/m^4	c_caBoundr
dflux_c_caBound_r_chdi	Diffusive flux, c_caBound, r component	mol/(m^2*s)	-Drr_c_caBound_chdi * c_caBoundr-Drz_c_caBound_chdi * c_caBoundz
grad_c_caBound_z_chdi	Concentration gradient, c_caBound, z component	mol/m^4	c_caBoundz
dflux_c_caBound_z_chdi	Diffusive flux, c_caBound, z component	mol/(m^2*s)	-Dzr_c_caBound_chdi * c_caBoundr-Dzz_c_caBound_chdi * c_caBoundz
grad_c_caBound_chdi	Concentration gradient, c_caBound	mol/m^4	sqrt(grad_c_caBound_r_chdi^2+grad_c_caBound_z_chdi^2)
dflux_c_caBound_chdi	Diffusive flux, c_caBound	mol/(m^2*s)	sqrt(dflux_c_caBound_r_chdi^2+dflux_c_caBound_z_chdi^2)
