# Supplementary figures and images for: Cell surface topology creates high Ca2+ signalling microdomains
Source: Cell Calcium. 2010 Apr;47(4):339–49. doi: 10.1016/j.ceca.2010.01.005 (PMC2877796; doi:10.1016/j.ceca.2010.01.005)

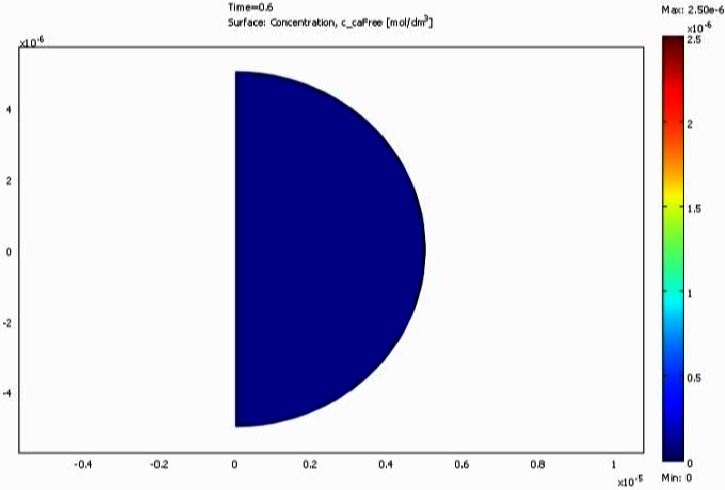

Supplement: Supplementary file 3 [file mmc3.jpg]

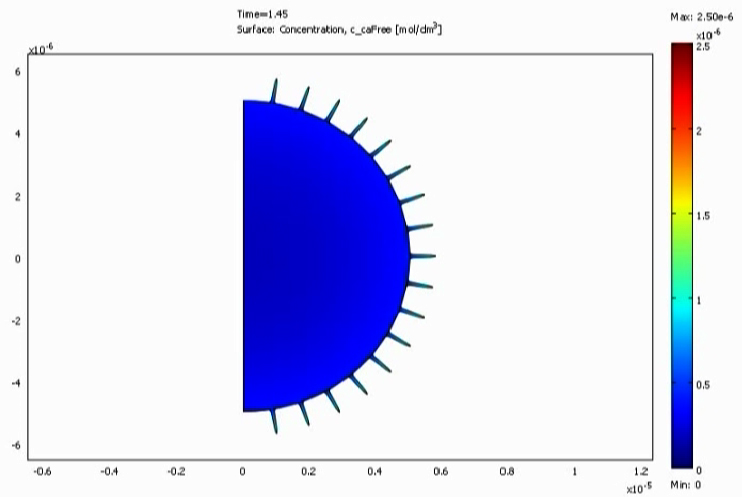

Supplement: Supplementary file 4 [file mmc4.jpg]

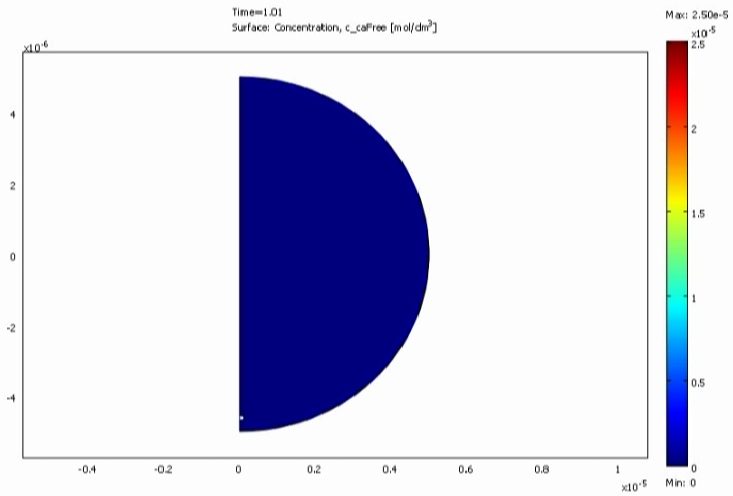

Supplement: Supplementary file 5 [file mmc5.jpg]

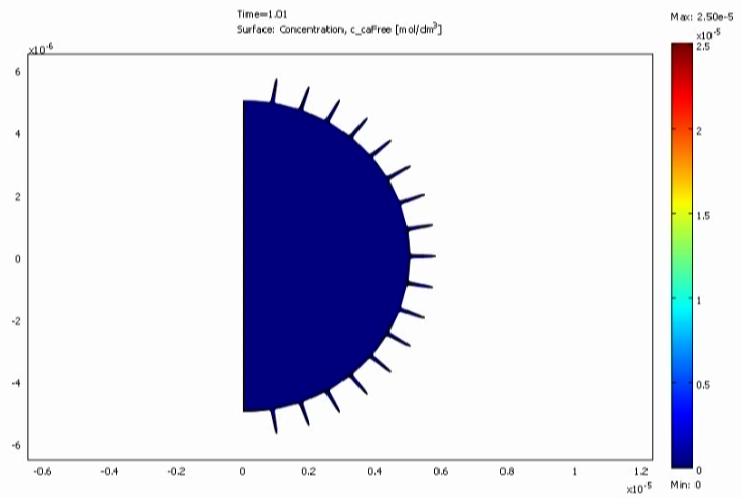

Supplement: Supplementary file 6 [file mmc6.jpg]
